# Agricultural adaptation in the native North American weed waterhemp, *Amaranthus tuberculatus* (Amaranthaceae)

**DOI:** 10.1371/journal.pone.0238861

**Published:** 2020-09-24

**Authors:** Katherine E. Waselkov, Nathaniel D. Regenold, Romy C. Lum, Kenneth M. Olsen

**Affiliations:** 1 Department of Biology, College of Arts and Sciences, Washington University, St. Louis, Missouri, United States of America; 2 Department of Biology, College of Science and Mathematics, California State University, Fresno, California, United States of America; NSW Department of Primary Industries, AUSTRALIA

## Abstract

There is increasing interest in documenting adaptation of weedy plant species to agricultural ecosystems, beyond the evolution of herbicide resistance. Waterhemp (*Amaranthus tuberculatus*) is a native plant of the Midwestern U.S. that began infesting agricultural fields in the 20^th^ century within the central portion of its range. We hypothesized that the vegetative growth and reproductive traits of waterhemp from this heavily infested central region provide differential fitness benefits in agricultural environments. We collected seeds from across the species’ native range, representing regions with varying degrees of waterhemp infestation, and planted them together in common garden soybean plots. A 2010 common garden experiment was conducted within the range of agriculturally weedy waterhemp (in Missouri), and a 2011 common garden experiment was conducted outside of this range (in Ohio). Days to flowering and flowering plant height, mature plant size data (height, number of branches, and length of the longest branch), and above-ground biomass were measured to estimate relative fitness. In both common garden locations, plants from regions where waterhemp occurs as an agricultural weed — including those from the heavily infested Mississippi Valley region (Iowa, Illinois, and Missouri) and the less severely infested Plains region (Nebraska, Kansas, and Oklahoma) — had higher relative performance in almost all fitness-related measures than plants from the Northeast region (Ohio, Michigan, and Ontario), which had little to no agriculturally weedy waterhemp at the time of our study. Further analysis revealed that fewer days to flowering in the Northeast populations can be largely accounted for by latitude of origin, suggesting a strong genetic influence on this reproductive trait. These findings suggest intraspecific variation in agricultural adaptation in a native U.S. weed, and support the use of agricultural weeds to study adaptation.

## Introduction

Agricultural weeds can be viewed as invasive plants of heavily human-modified environments, namely crop fields and rangelands [1]. Changing cultivation practices in the 20^th^ century, especially the intensification of farming since the 1940s, caused rapid shifts in crop field weed communities. Widespread adoption of conservation tillage, which leaves at least 30% of the soil surface covered by crop residue at planting time, has changed the species that are most problematic agriculturally relative to previous tillage systems [2, 3]. Furthermore, the introduction of herbicide-resistant crops in the 1990s, which by 2018 made up 94% of soybean and 90% of corn production in the U.S., led to increased reliance on herbicides and reduced tilling, and subsequently to shifts in the weed community in these fields [4–7].

Studies conducted since the 1970s have shown that many agricultural weeds contain adaptive genetic variation and may respond to selection (reviewed by [8]). However, studies of adaptation in agricultural weeds have focused primarily on the evolution of herbicide resistance (reviewed in [9–12]). Other than that single trait, the mechanisms permitting agricultural weed infestations, and the involvement of adaptive evolution in this process, remain largely unexamined. In particular, the role of intraspecific variation in agricultural adaptation is poorly understood [8, 1, 13]. Agricultural weeds cost an estimated $33 billion annually in the U.S. alone [14]; given their economic impact, it is surprising that adaptive evolution of weeds in traits other than herbicide resistance has not been more frequently studied. However, there has recently been increased interest in developing agricultural weeds as model systems for understanding rapid adaptation to human-altered habitats, for example in sunflowers [15, 16], morning glory [17, 18], radish [19, 20], and weedy rice [21].

Like invasive species of natural habitats, agricultural weeds may contain high levels of genetic variation due to multiple introductions or origins, and they may be locally adapted to different environmental conditions in agroecosystems (e.g. [15, 22–25]). An agricultural weed may also be native to the geographical region where it invades agroecosystems: many weed species were originally pioneers in naturally disturbed habitats, before making the ecological and/or evolutionary leap to crop fields [26]. Both native and introduced weeds often occur outside agricultural fields, in natural environments or as ruderal weeds of railroads and roadsides, leading to opportunities for possible gene flow and/or selection in different habitat types across a landscape [8, 27].

The focus of this study, waterhemp (*Amaranthus tuberculatus* (Moq.) Sauer), is an herbaceous, dioecious, wind-pollinated annual plant native to the Midwestern U.S., where it occurs naturally along riverbanks and in floodplains [28]. It has increasingly been observed in agricultural habitats since the 1950s and has become a major problem for farmers since the 1990s [29, 30]. In Illinois alone, waterhemp accounts for about 10% of weed control costs for corn and soybean fields, costing farmers an additional $65 million per year (Patrick Tranel, Univ. of IL, 2013 pers. comm.). If uncontrolled, it can reduce corn yields by up to 74%, and soybean yields by as much as 56% [31]. As a small-seeded annual with germination throughout the growing season, waterhemp is a prime example of the class of agricultural weeds that benefited from the widespread adoption of conservation tillage in the late 20^th^ century [6, 32, 33]. Rapid evolution of herbicide resistance has also contributed greatly to waterhemp’s success. To date, resistance to seven different chemical classes of herbicides has been detected in *A*. *tuberculatus* populations [34–40]. Additionally, many populations exhibit resistance to multiple chemical classes [41].

Since waterhemp is a Midwestern native with a very broad geographic range, which has only recently invaded agricultural ecosystems and has demonstrably evolved in response to changing agricultural practices (i.e., via herbicide resistance mutations), it is an ideal candidate for the study of intraspecific variation in other, non-herbicide-related phenotypic traits that facilitate adaptation in agricultural weeds [42]. Previous studies have examined herbicide resistance and other fitness components of waterhemp within agricultural fields with the ultimate goal of weed control [43–45], and one study examined fitness differences between several agriculturally weedy populations in a common garden with no crops present [46]. However, no previous work has explicitly compared the relative fitness of a broad sampling of populations from across the species’ native range, collected in natural habitats (rather than agricultural habitats), within an agricultural setting.

**Fig 1:**
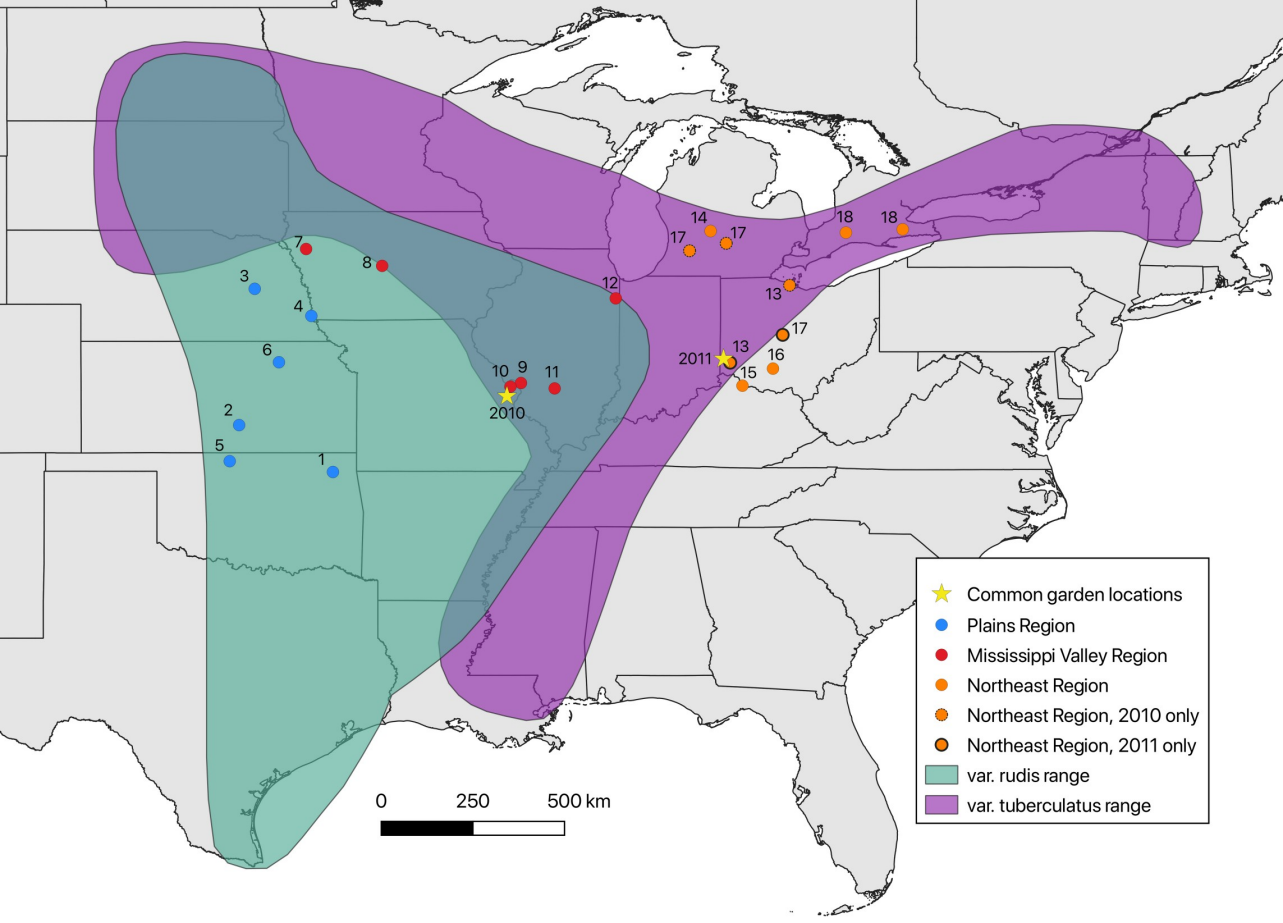
Source populations and common garden locations within the geographical range of *Amaranthus tuberculatus* s.l. (waterhemp). The historical range of *A*. *tuberculatus* var. *rudis* is shaded in green, and range of *A*. *tuberculatus* var. *tuberculatus* in purple, with the opaque green shading showing the areas of overlap between the varieties (adapted from [22]). Source populations for seeds are shown as circles: six blue circles = Plains region, six red circles = Mississippi Valley region, and ten orange circles = Northeast region. Few seeds were available for some Northeast region populations, and so in two cases, two geographically proximate seed collections were combined for the common garden experiments. Three orange circles with dashed outlines represent populations that were only included in 2010; two orange circles with bold outlines represent populations that were only included in 2011. Common garden locations are shown as yellow stars. United States basemap from the U.S. Census Bureau (public domain); Canadian basemap from Statistics Canada (public domain).

Several additional observations lend credence to the hypothesis that waterhemp populations may vary in their potential for success in agroecosystems. While *A*. *tuberculatus* naturally ranges from the Great Plains to southern Ontario, the region of agriculturally-problematic waterhemp is smaller; it is a major cause of crop yield loss in parts of Iowa, Missouri, Illinois, and Indiana, whereas as of 2010, it was an opportunistic weed in the Plains region (the Dakotas, Nebraska, Kansas, Oklahoma, Texas), and was not known to occur agriculturally in most of Ohio (outside of a handful of western counties; J. Stachler, pers. comm.) and had only just begun to invade Ontario [30, 47] (Fig 1).

Prior to a recent taxonomic study [48], waterhemp was divided into two species based mainly on fruit dehiscence and geographic range: *A*. *tuberculatus*, the indehiscent-fruited taxon found to the east of the Mississippi River; and *A*. *rudis* (earlier misapplied name = *A*. *tamariscinus*; see [49]), the dehiscent-fruited taxon found most frequently west of the Mississippi River, and commonly considered the "weedy" form of waterhemp [29, 49, 50] (Fig 1). Some authors still distinguish the two former species as varieties: *A*. *tuberculatus* var. *tuberculatus* and var. *rudis* [51]; this taxonomy is used in this paper, with the entire species referred to as *A*. *tuberculatus* sensu lato (s.l.). Furthermore, a range-wide population genetic study using 10 microsatellite markers, conducted in tandem with the present study, revealed two genetically differentiated subpopulations within *A*. *tuberculatus* that correspond to the western and eastern portions of the species range [52]. While these genetic subgroups are broadly demarcated by the Mississippi River, some populations east of the Mississippi, in areas that have been invaded by agricultural waterhemp, show genetic affinity to the western group. A recent population genomic study based on SNP data has also indicated that agriculturally weedy waterhemp is strongly associated genetically with the “western” subpopulation [42]. Together these findings suggest that waterhemp from the “western” subpopulation may have higher fitness as an invasive agricultural weed.

In this study, we conducted two common garden experiments to test the hypothesis that the vegetative growth and reproductive traits of waterhemp from the region with the highest level of agricultural infestation provide differential fitness benefits in agricultural environments. Our experimental setup consisted of planting waterhemp sampled from populations across the species range (nearly all collected from natural habitats) into agricultural field plots, in a common garden study design. Field plots were planted with soybeans to provide a crop “matrix” for the weeds, because waterhemp is the most problematic weed for this crop in the Midwest. Two different common garden experiments were conducted, one inside of the area of agriculturally-problematic waterhemp (in Missouri), and one experiment outside this area (in Ohio). We excluded herbicides from the study design, and instead focused on collecting data for several different measures of vegetative growth related to total plant size (flowering plant height, mature plant dimensions, and biomass) and one reproductive life history trait (days to flowering), across the growing season to obtain a broad assessment of the relative fitness of waterhemp from different geographical regions. For the purposes of the common garden experiments, the geographical range was divided into three regions, where populations were hypothesized to have three different levels of adaptation to agricultural environments: the Plains region (including KS, NE, and OK populations in the experiment), the Mississippi Valley region (including MO, IA, and IL), and the Northeast region (including OH, MI, and ON) (Fig 1). We specifically asked the following question: Is waterhemp from the most heavily infested states (Missouri, Illinois, and Iowa, which we call the “Mississippi Valley” region) more fit in soybean fields than waterhemp from a less heavily infested region (the U.S. Plains states) and a largely uninvaded region (the Northeastern U.S. and Ontario)? We hypothesized that Mississippi Valley region waterhemp populations would exhibit adaptation to agricultural conditions, and from this hypothesis, we predicted that Mississippi Valley region plants would have higher relative fitness than plants from other regions in both common gardens.

## Materials and methods

The following section is a concise summary of our methods; for a more detailed version, see the S1 Methods and Results.

### Seed collection

In the fall of 2009 and 2010, seeds were collected from populations across the range of *A*. *tuberculatus* s.l. for use in common garden experiments in two locations: Missouri (summer 2010) and Ohio (summer 2011). For both common garden experiments, six populations from each of the three different geographic regions (see above) were selected for the experiment (Fig 1). Almost all populations were sampled from natural habitats rather than agricultural fields, with the exception of one Iowa population (Population 7), and one Illinois population (Population 12), which were included to maximize geographical representation. Population structure analyses indicate there is no genetic differentiation between agricultural and natural populations on the geographical scale of central U.S. states [52]; however, to test for a possible effect of including these agricultural populations, data were analyzed with and without Populations 7 and 12 (see Data analysis, below). Seeds from the two Iowa populations were obtained from the USDA GRIN database: they were originally collected in 1989 and 1996 by D. Pratt, and seed stocks have been maintained by the USDA without being grown out since collection (D. Brenner, pers. comm.).

For the two years of the common garden experiments (2010, 2011), the same source populations were used to represent the Plains region and the Mississippi Valley region. For the Northeast region, two newly-collected Ohio populations were included in 2011 (and two previous Northeastern populations from 2010 were dropped) to represent the portion of the state where agricultural fields have been infested by waterhemp, and to attempt to correct for possible confounding of latitude of origin with agricultural adaptation (see Table 1 and Fig 1). Voucher specimens for each population were deposited at the Missouri Botanical Garden (MO) herbarium. By morphological criteria, the Plains and Mississippi Valley populations were mostly *A*. *tuberculatus* var. *rudis*, whereas the Northeast populations were mostly *A*. *tuberculatus* var. *tuberculatus* (Table 1).

**Table 1:** Populations of *Amaranthus tuberculatus* used common garden experiments.

Region of origin	Population number (common garden year)	State or province of collection	Utricle dehiscence/Taxonomic variety	Population GPS location
Plains	1	OK	Dehiscent/var. *rudis*	N 36.48063°, W 95.25453°
Plains	2	KS	Immature/unknown	N 37.74475°, W 97.78386°
Plains	3	NE	Dehiscent/var. *rudis*	N 41.41872°, W 97.36353°
Plains	4	NE	Dehiscent/var. *rudis*	N 40.68752°, W 95.83635°
Plains	5	OK	Dehiscent/var. *rudis*	N 36.77166°, W 98.03800°
Plains	6	KS	Dehiscent/var. *rudis*	N 39.43923°, W 96.71025°
Mississippi Valley	7*	IA (USDA PI 603872)	Dehiscent/var. *rudis*	N 42.49167°, W 95.97795°
Mississippi Valley	8	IA (USDA PI 553086)	Dehiscent/var. *rudis*	N 42.03696°, W 93.92773°
Mississippi Valley	9	MO	Immature/unknown	N 38.87902°, W 90.18393°
Mississippi Valley	10	MO	Dehiscent/var. *rudis*	N 38.78155°, W 90.46896°
Mississippi Valley	11	IL	Immature/unknown	N 38.73371°, W 89.27585°
Mississippi Valley	12*	IL	Indehiscent/var. *tuberculatus*	N 41.16075°, W 87.62755°
Northeast	13 (2010)	OH	Indehiscent/var. *tuberculatus*	N 41.51450°, W 82.93943°
Northeast	13 (2011)	OH	Dehiscent/var. *rudis*	N 39.42743°, W 84.54071°
Northeast	14	MI	Indehiscent/var. *tuberculatus*	N 42.97538°, W 85.07140°
Northeast	15	OH	Indehiscent/var. *tuberculatus*	N 38.80763°, W 84.21171°
Northeast	16	OH	Indehiscent/var. *tuberculatus*	N 39.26801°, W 83.38861°
Northeast	17 (2010)	MI/MI (seeds from 2 sites pooled)	Indehiscent/var. *tuberculatus*	N 42.64500°, W 84.64970°; N 42.44408°, W 85.63737°
Northeast	17 (2011)	OH	Dehiscent/var. *rudis*	N 40.17745°, W 83.12640°
Northeast	18	ON/ON (seeds from 2 sites pooled)	Indehiscent/var. *tuberculatus*	N 43.02070°, W 79.89105°; N 42.93375°, W 81.42106°

**Fig 2:**
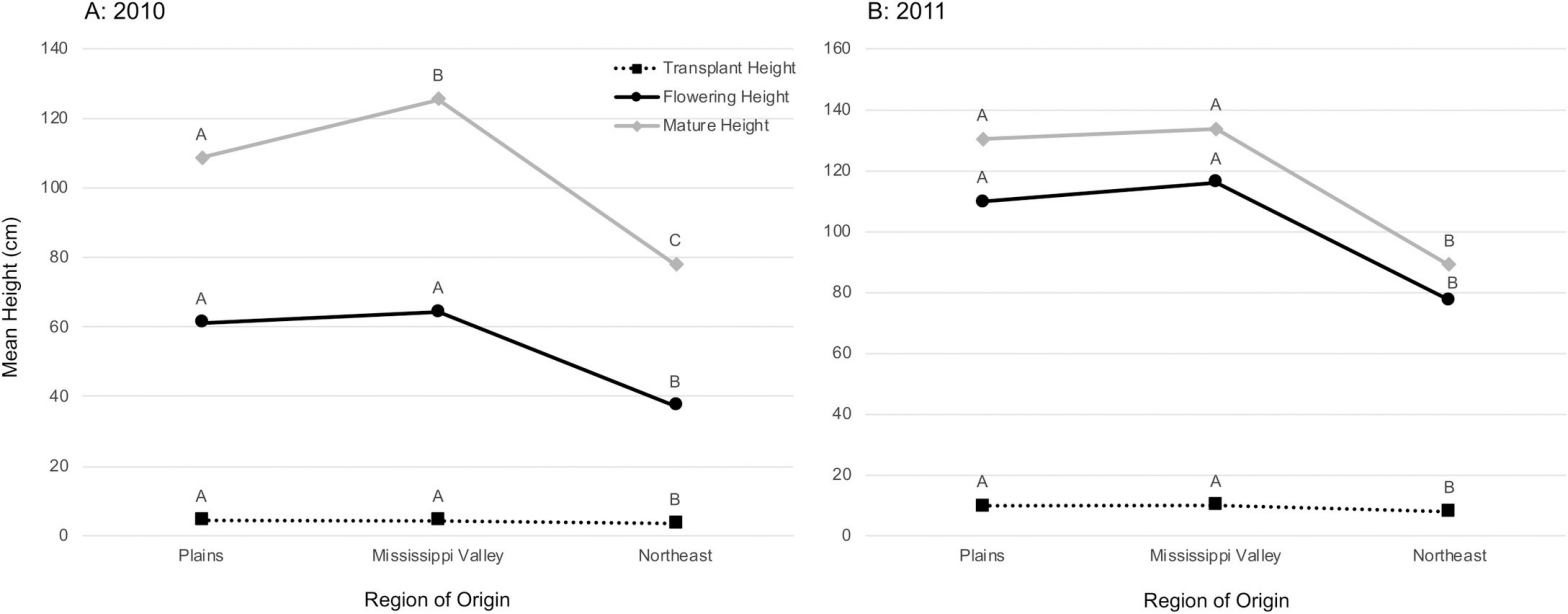
Line graphs of mean height (cm) over time, by region of origin. Letters next to points represent groups that are significantly different (different letters) or are not significantly different (same letters) as determined by post-hoc tests. A = 2010, B = 2011.

**Fig 3:**
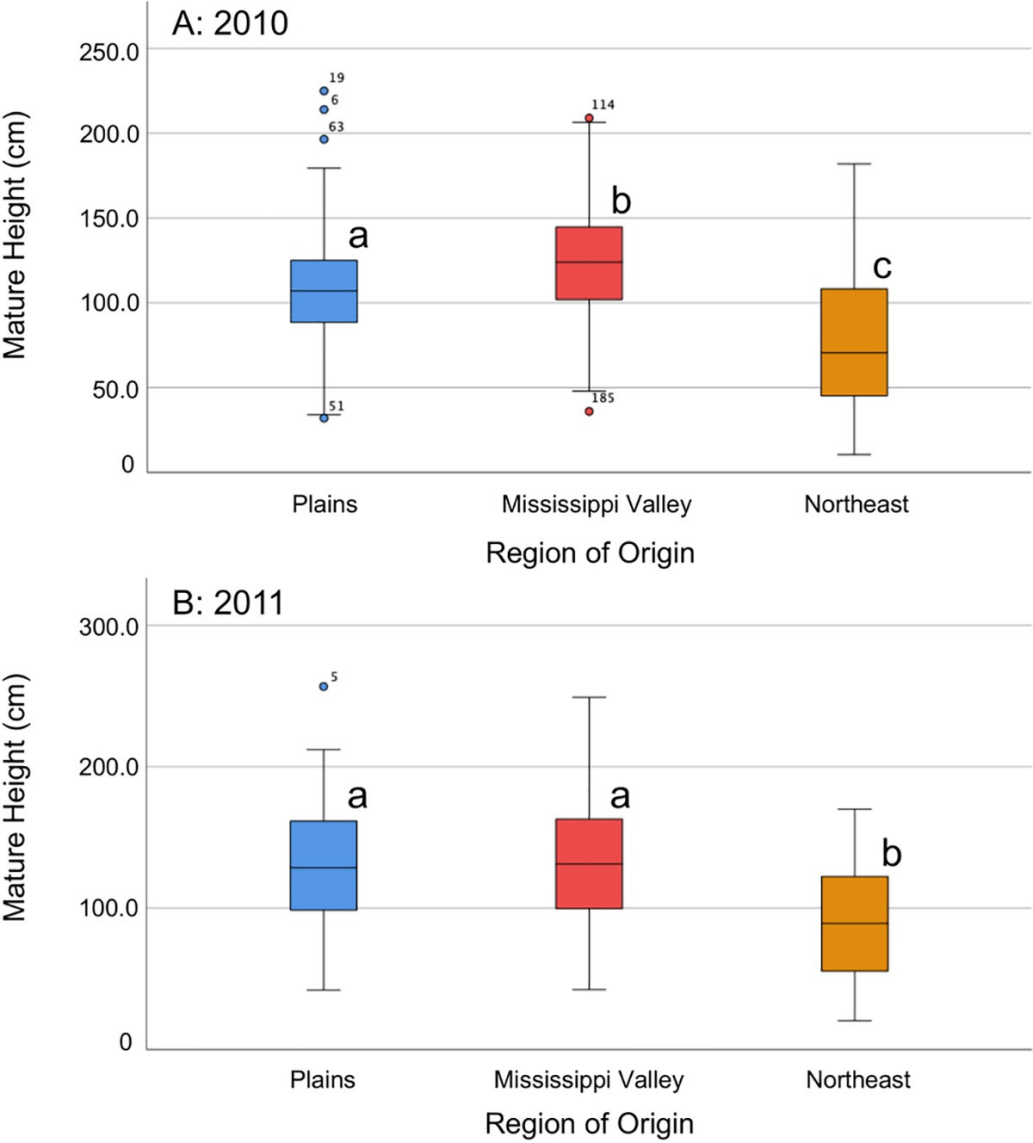
Box plots of mature height (cm) over time, by region of origin. Letters next to box plots represent groups that are significantly different (different letters) or are not significantly different (same letters) as determined by post-hoc tests. Circles represent outliers (cases with values between 1.5 and 3 times the interquartile range). A = 2010, B = 2011.

**Fig 4:**
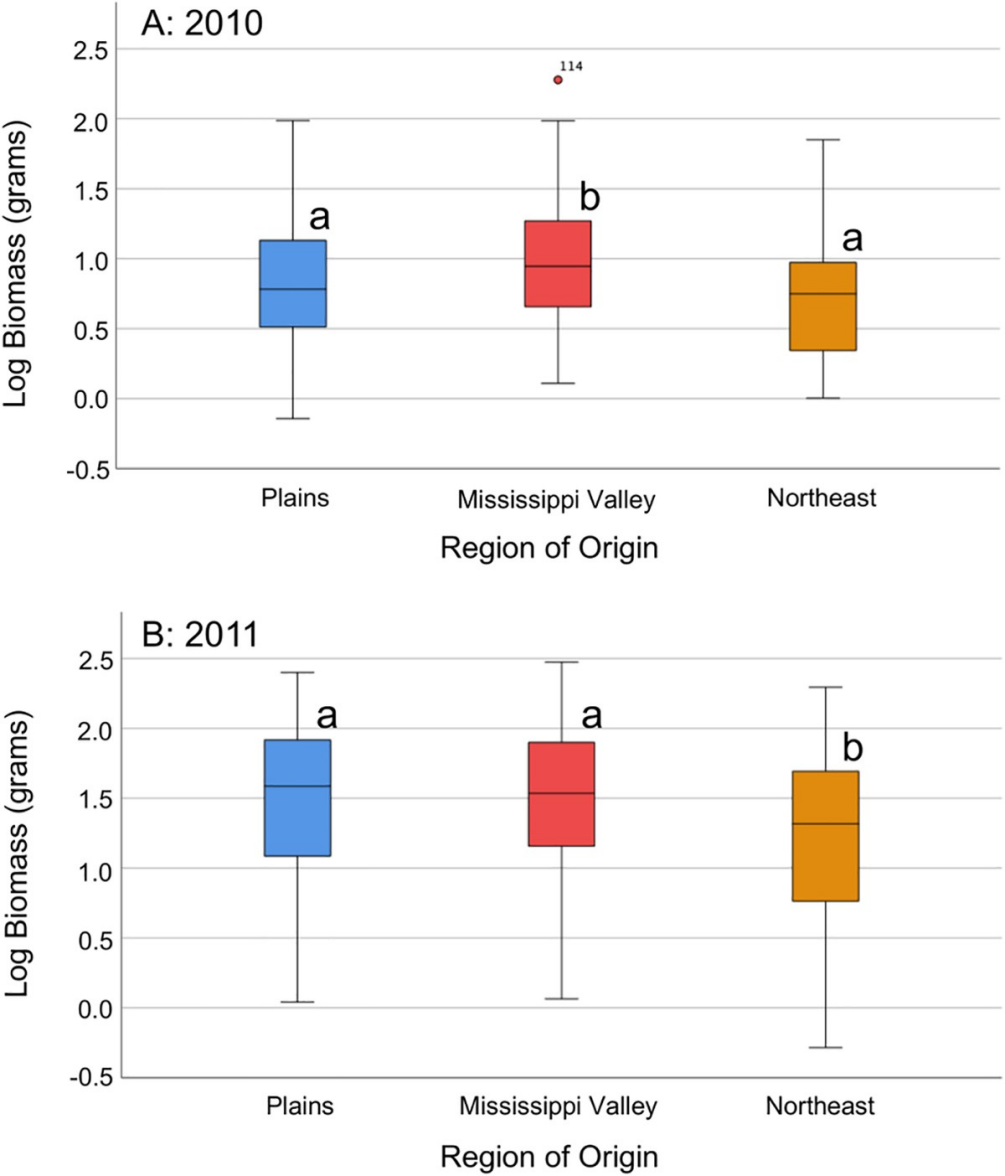
Box plots of log_10_ dry above-ground biomass, by region of origin. Letters next to box plots represent groups that are significantly different (different letters) or are not significantly different (same letters) as determined by post-hoc tests. Circles represent outliers (cases with values between 1.5 and 3 times the interquartile range). A = 2010, B = 2011.

**Fig 5:**
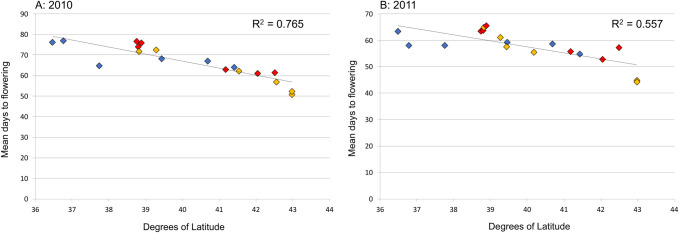
Scatterplots of mean days to flowering by latitude of origin (i.e., population, rearranged by increasing latitude). Trendline and linear R^2^ value calculated using SPSS. Blue points = Plains populations, red points = Mississippi Valley populations, gold points = Northeast populations. A = 2010, B = 2011.

All seeds collected for the present study, except for populations 7 and 8 (USDA PI numbers refer to GRIN accession numbers). Utricle dehiscence refers to the condition of the fruit on the female voucher specimen collected for each population, from which the taxonomic variety was inferred. Region and population numbers correspond to Figs 2 to 5 and all supplementary data tables and figures.

* = populations that were collected originally in agricultural fields.

### Common garden setup

The common garden design was as follows: each year, three replicate plots were established at one location (Washington University’s Tyson Research Center in Eureka, MO in 2010, and Miami University’s Ecological Research Center in Oxford, OH in 2011). Logistical constraints prevented conducting these very large field experiments in both locations in the same year. Therefore, we chose to maximize our ability to observe potential differences in the behavior of populations from across the range, based on common garden location within or outside of the region of highest infestation, at the expense of temporal replication within a location. Each year, the three replicate plots had dimensions of 7x10 m, and are referred to as “blocks” in the statistical analyses and in all subsequent mentions in the paper. Each block was planted with RoundUp Ready soybeans suitable for each location (Missouri: Asgrow RR3830 variety; Ohio: Genuity Star RR3404 variety), at a density of 160,000 plants/acre, from 19-26 May 2010 and on 8 June and 25 June 2011. The soybeans rows provided an agricultural “matrix” to compete against the weedy waterhemp, which was the focus of our hypotheses; therefore, no data were collected from the soybeans themselves. Because of an unusually wet spring in Ohio, soybean planting was delayed in 2011 compared to the previous year.

Waterhemp seeds were stratified in cool moist conditions (4ºC) for 3-4 months before planting. Waterhemp seedlings were started in the Washington University greenhouse on May 19 (2010) or June 10 (2011), timed to coincide with soybean planting in both years. An average of ~3 seeds/maternal waterhemp plant were germinated, from 10 parent plants per population, and six populations representing each of the three geographical collection regions. Just prior to transplantation, seedlings were sorted into three groups of 180 plants each (one seedling/parent and 10 seedlings/population), randomly assigned a number from 1-540, and then arranged in numerical order in these sets of 180, for each block in the common garden. Each individual plant was treated as one experimental unit statistically. There is some evidence in the genus *Amaranthus* that there are strong maternal effects on seed dormancy/germination [53, 54]; therefore, the height of each seedling was recorded just prior to transplanting, to use as a control for maternal effects on early growth. The 3-4 week old waterhemp seedlings were transplanted into the soybean blocks from 16-19 June 2010 and from 6-8 July 2011, into 13 five-meter long rows with 13 plants per row and a 14^th^ row with 11 plants. The blocks were hand weeded throughout the growing season to remove all plants other than soybeans and waterhemp.

### Plant measurements

In 2010, starting a few days after transplanting and every week thereafter, plant survivorship in the common gardens was recorded. In 2010, flowering start date, flowering plant height, and sex of the plant was recorded from the beginning of flowering on 29 June to 19 August, every 5-9 days. In 2011, flowering start date, flowering plant height, and sex of the plant were recorded every 2-3 days, from 11 July-19 August. In both years, an open flower on a male or female plant was taken as the start of flowering, and days to flowering was measured as the number of days between planting and the start of flowering. In 2010, mature plant measurements were taken when ~75% of flowers were open (for male plants, which grow very little once flowering begins) or ~75% of flowers had set seed (for female plants, which continue to grow after flowering begins), between 13 August and 5 October as plants matured asynchronously, approximately every 2-3 weeks. Mature height, number of branches off the main stem, and length of the longest primary branch were recorded for each waterhemp plant.

In 2011, because the geographical area around Oxford, OH did not yet have a problem with agricultural waterhemp at that time, procedures were implemented to contain gene flow from the experimental waterhemp into surrounding agricultural fields and/or nearby riverbank populations. To prevent the pollen from being dispersed by wind, male plants were measured for mature data and harvested as soon as their first flower opened, every two-three days from 15 June to 2 September. Female plants were measured for mature height, number of branches, and length of the longest branch and harvested at approximately the same point as in the Missouri common garden (every 2-3 weeks from 2 September to 12 October), before many seeds/fruits could drop from the plant.

Immediately after mature measurements were taken, the plant’s above-ground biomass was removed at ground level, placed in a brown paper bag, and dried in a Conviron plant growth chamber (PGW36 model, Conviron, Winnipeg, Manitoba, Canada) set at 38ºC in the Washington University greenhouse. Batches of bags were left in the chamber for 9-15 days, at which time each bagged plant was weighed on an electronic scale. Dried above-ground biomass measurements were recorded to the nearest 0.01 g, and the average weight of five empty bags was subtracted from the raw biomass measurement.

### Data analysis

All plant data were analyzed using IBM SPSS Statistics 1.0.0.1213 (IBM Corp., 2018). Each year’s data were analyzed separately, as was each measured response variable. First, all continuous data were tested for normality using the Shapiro-Wilk test. If the data were not normal, they were either log_10_ transformed or square-root transformed. Analyses were run with and without outliers (detected using SPSS box plots). Nonparametric tests were used for ordinal data (days to flowering).

A univariate general linear model (GLM) or the equivalent nonparametric test was used to analyze each dataset, including height at transplantation, flowering plant height, mature plant height, branch number, length of the longest branch, dry above-ground biomass, and days to flowering. Additionally, a repeated-measures general linear model was used to analyze height over time. Multivariate GLM was also used to analyze mature plant height, branch number, longest branch length, and dry above-ground biomass together, because of the correlation of these mature plant measurements (moderate positive correlation (0.3-0.85) verified with a Pearson’s correlation matrix). Height at transplantation was included as a covariate in all general linear models (with the exception of the repeated measures GLM for height, where it was the first time point in the dataset), to control for maternal effects. The fixed factors in each GLM were geographical region of origin (Plains, Mississippi Valley, and Northeast) and sex of the plant. The random factors were block and population nested within region, and the intercept was included in the model. Interactions between sex, region, and block were included in the models initially, but were omitted for the final analyses as these interaction terms never had a significant effect on the dependent variables (results not shown). For significant results for continuous dependent variables, post-hoc Tukey HSD tests were used to determine whether means were significantly different between each pair of regions and populations. When the results of Levene’s test of equality of error variances were significant for continuous data, or when a covariate was included, pairwise comparison of estimated marginal means (with Bonferroni correction of significance values) was performed in lieu of Tukey HSD tests. Dunn’s multiple comparison tests with Bonferroni correction of significance values were performed post hoc for ordinal data.

Because the growing conditions differed substantially between years, the datasets from Missouri and Ohio common gardens were analyzed separately (with no attempt made to combine data across years). Additionally, to rule out any confounding factors introduced by harvesting the Ohio male plants earlier than the Missouri males, only female data were analyzed for both common gardens and compared to the full data set. To rule out possible bias introduced by including the two agricultural populations (7 and 12), data were analyzed with and without these populations included. Finally, to further assess potential outlier effects of high latitude populations on the response variables, all analyses were run with the omission of data from populations 14 and 18 (the two populations collected at the highest latitudes, both found in the Northeast region) and compared to the full data set.

## Results

### General observations

Seed germination after stratification took place over a period of about 2 weeks: the germination rate for most populations was >90% by this time point. The remaining seeds either never germinated (and were suspected to be inviable), or germinated later but were not used in the experiments.

Mortality after establishment and before maturity was minimal in both years. In total, 449 of 540 plants survived the transplantation period in 2010, and 14 of these established plants died during the growing season. Mortality in 2010 stemmed almost entirely from a “damping off” fungal infection that killed the plants within 10 days of transplantation, without regard for geographic region of origin. In 2011, 519 plants out of 540 survived transplantation, and only one of these survivors died during the growing season. Because some plants died after flowering but before maturity, the number of individuals measured for flowering data vs. mature data differed slightly in both years (S1 Table). In both years, analyses with and without early-flowering/early-dying/damaged plants (see Methods) had generally consistent results, with lower significance for the datasets with these plants removed (probably because of lower sample sizes); to be conservative, results for the latter datasets are reported below. Also, analyses run with and without statistical outliers were qualitatively similar (in terms of statistical significance); therefore, for all analyses, only results with outliers included are presented.

Sex was frequently significant in the GLM analyses of the full dataset; female plants were taller on average at maturity, heavier, and took more days to flower, and they had more branches (and longer longest branches in 2011) than male plants did, regardless of region or population (Table 2; S1 Table vs. S3 Table). Sex ratios in our experimental design could not be controlled (as at the time of this study, male vs. female waterhemp plants could only be distinguished once flowering had begun; [55]). Sex ratios differed between years and regions. The 2010 experiment had female-biased data: the male:female ratios, averaged over different types of measurements, were 0.87 for the Plains region, 0.93 for the Mississippi Valley region, and 0.79 for the Northeast region. The 2011 common garden data were male-biased for all measurements. In 2011, the male:female ratios, averaged over different types of measurements, were 1.19 for the Plains region, 1.65 for the Mississippi Valley region, and 1.77 for the Northeast region. The difference between years is partially explained by the fact that the Ohio males were harvested earlier than the Missouri males, and some of the Missouri males had senesced by harvest time. However, the number of females from any particular region was very similar between years (S1 Table); for this reason, results of data analysis for only female plants are reported below, in addition to the full dataset.

**Table 2:** Results from general linear models (GLM) or nonparametric Kruskal-Wallis tests of the effect of fixed and random factors on transplant height, flowering height, mature height, mature branch number, length of longest mature branch, dry above-ground biomass, and days to flowering, for the full dataset.

GLM	2010 Transplant Height^a^			2011 Transplant Height		
Factor	df (Hypothesis, Error)	F ratio	P-value	df (Hypothesis, Error)	F ratio	P-value
Intercept	1, 16.776	5457.063	**<0.001**	1, 15.442	773.815	**<0.001**
Region	2, 15.981	7.384	**0.005**	2, 15.138	3.795	**0.046**
Population (Region)	15, 260	2.228	**0.006**	15, 382	6.146	**<0.001**
Sex	1, 260	0.952	0.330	1, 382	0.453	0.501
GLM	2010 Flowering Height^b^			2011 Flowering Height^b^		
Factor	df (Hypothesis, Error)	F ratio	P-value	df (Hypothesis, Error)	F ratio	P-value
Intercept	1, 36.213	30.649	**<0.001**	1, 17.326	162.072	**<0.001**
Region	2, 15.490	5.023	**0.021**	2, 15.188	7.042	**0.007**
Population (Region)	15, 257	6.832	**<0.001**	15, 379	10.885	**<0.001**
Block	2, 257	15.971	**<0.001**	2, 379	10.669	**<0.001**
Transplant Height	1, 257	2.649	0.105	1, 379	0.967	0.326
Sex	1, 257	56.491	**<0.001**	1, 379	2.774	0.097
GLM	2010 Mature Height^b^			2011 Mature Height^b^		
Factor	df (Hypothesis, Error)	F ratio	P-value	df (Hypothesis, Error)	F ratio	P-value
Corrected Model	21	8.411	**<0.001**	21	35.401	**<0.001**
Intercept	1	57.922	**<0.001**	1	531.375	**<0.001**
Region	2, 249	39.872	**<0.001**	2, 367	78.560	**<0.001**
Population (Region)	15, 249	3.577	**<0.001**	15, 367	9.638	**<0.001**
Block	2, 249	12.269	**<0.001**	2, 367	14.847	**<0.001**
Transplant Height	1, 249	0.207	0.649	1, 367	0.881	0.348
Sex	1, 249	13.769	**<0.001**	1, 367	279.813	**<0.001**
GLM	2010 Mature Branch Number^a^^,^^b^			2011 Mature Branch Number^a^^,^^b^		
Factor	df (Hypothesis, Error)	F ratio	P-value	df (Hypothesis, Error)	F ratio	P-value
Corrected Model	21	9.625	**<0.001**	21	18.377	**<0.001**
Intercept	1	90.801	**<0.001**	1	1045.315	**<0.001**
Region	2, 249	20.955	**<0.001**	2, 367	13.566	**<0.001**
Population (Region)	15, 249	3.843	**<0.001**	15, 367	6.592	**<0.001**
Block	2, 249	7.591	**0.001**	2, 367	5.550	**0.004**
Transplant Height	1, 249	0.622	0.431	1, 367	4.266	**0.040**
Sex	1, 249	98.153	**<0.001**	1, 367	187.272	**<0.001**
GLM	2010 Length of Longest Mature Branch^a^^,^^b^			2011 Length of Longest Mature Branch^a^^,^^b^		
Factor	df (Hypothesis, Error)	F ratio	P-value	df (Hypothesis, Error)	F ratio	P-value
Corrected Model	21	1.806	**0.019**	21	23.761	**<0.001**
Intercept	1	32.975	**<0.001**	1	697.764	**<0.001**
Region	2, 249	1.279	0.280	2, 367	25.071	**<0.001**
Population (Region)	15, 249	1.091	0.365	15, 367	7.819	**<0.001**
Block	2, 249	9.235	**<0.001**	2, 367	25.324	**<0.001**
Transplant Height	1, 249	0.520	0.471	1, 367	1.067	0.302
Sex	1, 249	0.100	0.752	1, 367	203.772	**<0.001**
GLM	2010 Dry Above-ground Biomass^b^^,^^c^			2011 Dry Above-ground Biomass^b^^,^^c^		
Factor	df (Hypothesis, Error)	F ratio	P-value	df (Hypothesis, Error)	F ratio	P-value
Corrected Model	21	7.074	**<0.001**	21	36.473	**<0.001**
Intercept	1	26.911	**<0.001**	1	528.780	**<0.001**
Region	2, 249	13.228	**<0.001**	2, 367	29.871	**<0.001**
Population (Region)	15, 249	2.368	**0.003**	15, 367	10.811	**<0.001**
Block	2, 249	13.062	**<0.001**	2, 367	30.560	**<0.001**
Transplant Height	1, 249	0.024	0.876	1, 367	0.412	0.521
Sex	1, 249	65.790	**<0.001**	1, 367	364.582	**<0.001**
Kruskal-Wallis Test	2010 Days to Flowering^d^			2011 Days to Flowering^d^		
Factor	df	Chi-squared statistic	P-value	df	Chi-squared statistic	P-value
Region	2	12.237	**0.002**	2	11.542	**0.003**

Significant values at alpha = 0.05 are bold.

^a^Square-root transformed data

^b^With square-root transformed transplant height as a covariate (2010), or transplant height as a covariate (2011)

^c^Log transformed data

^d^Categorical data

### Height over time

Repeated measures analyses of longitudinal height data (transplant, flowering, and mature heights) showed that the interaction between time and region of origin was highly significant in both of the common garden experiments (Table 3). General linear models on each time point independently also show a significant effect of region at all time points (Fig 2). Even before the waterhemp was placed in the soybean plots, there was a significant effect of region on height at transplantation in 2010 (F_2,15.981_=7.384, P=0.005) and in 2011 (F_2,15.138_=3.795, P=0.046; Table 2). Posthoc tests (Tukey HSD or Bonferroni-corrected pairwise comparison of estimated marginal means) showed that plants from the Northeastern region were on average shorter than plants from the other two regions (P_1,3_<0.001 and P_2,3_= 0.002 for 2010; P_1,3_ and P_2,3_<0.001 for 2011). The magnitude of this regional height difference increased as the plants grew, with Northeastern plants’ average flowering height being significantly shorter in 2010 (F_2,15.490_=5.023, P=0.021) and 2011 (F_2,15.188_=7.042, P=0.007; posthoc tests, P_2,3_ and P_1,3_ <0.001 for both years). Mature Northeastern plants were also significantly shorter in both years (2010, F_2,249_=39.872, P<0.001; 2011, F_2,367_=78.560, P<0.001; Fig 3). In the 2010 common garden, Mississippi Valley plants were the tallest at maturity (posthoc tests, P_1,2_, P_2,3_ and P_1,3_<0.001), but in 2011, there was no difference between mature heights of Mississippi Valley and Plains plants (P_1,2_= 0.145, P_2,3_ and P_1,3_<0.001).

**Table 3:** Results from repeated measures general linear models (GLM) for the effect of independent variables on height over time (within-subjects effects), or multivariate GLMs for the effect of independent variables on multivariate mature data.

GLM	2010 Height over Time			2011 Height over Time		
Factor	df (Hypothesis, Error)	F ratio	P-value	df (Hypothesis, Error)	F ratio	P-value
Time	1.755, 452.904	1508.880	**<0.001**	1.425, 541.522	5045.498	**<0.001**
Time*Region	3.511, 452.904	29.886	**<0.001**	2.850, 541.522	73.567	**<0.001**
Time*Population (Region)	26.332, 452.904	3.683	**<0.001**	21.376, 541.522	8.909	**<0.001**
Time*Block	3.511, 452.904	9.228	**<0.001**	2.850, 541.522	12.843	**<0.001**
Time*Sex	1.755, 452.904	22.135	**<0.001**	1.425, 541.522	229.748	**<0.001**
GLM	2010 Multivariate Mature Data^a^			2011 Multivariate Mature Data^a^		
Factor	df (Hypothesis, Error)	F ratio	P-value	df (Hypothesis, Error)	F ratio	P-value
Intercept	4, 246	29.230	**<0.001**	4, 364	296.708	**<0.001**
Region	8, 494	12.425	**<0.001**	8, 730	27.530	**<0.001**
Population (Region)	60, 996	2.393	**<0.001**	60, 1468	3.971	**<0.001**
Block	8, 494	11.558	**<0.001**	8, 730	9.017	**<0.001**
Transplant Height	4, 246	0.537	0.709	4, 364	3.949	**0.004**
Sex	4, 246	66.019	**<0.001**	4, 364	104.316	**<0.001**

Greenhouse-Geisser test results reported for all repeated measures within-subjects tests; Pillai’s Trace test results reported for all multivariate statistics. Significant values at alpha = 0.05 are bold.

^a^With square-root transformed transplant height as a covariate (2010), or transplant height as a covariate (2011).

Population (nested within region) was also significant for all of these height analyses (Table 2). Population-level results for mature height were examined, as the GLMs indicated that maturity was likely to show the greatest differences out of the three time points. In 2010, several populations from the Plains and Mississippi Valley regions (1, 9, 10, and 11) were significantly taller than the shortest populations (13, 14, 17, and 18, all from the Northeast; S1A Fig). In 2011, again, many of the Plains and Mississippi Valley populations (1, 4, 6, 9, 10, and 11) were significantly taller than four out of six Northeast populations (13, 14, 16, and 18), and the Northeast populations 14 and 18 were significantly shorter than all other populations (S1B Fig). Block always had a significant effect on flowering and mature heights (Table 2); however, given the lack of significant interactions between block and region, block-by-block results were not examined.

Sex was also significant in several of the height univariate GLMs (Table 2). With only female data, transplant height is not quite significant in 2011 (F_2,17.173_=3.213, P=0.065), probably due to smaller sample size in each region, as the posthoc relationships between regions remain the same as in the full dataset (S2 and S3 Tables). In both years, female-only flowering height (2010: F_2,16.557_=4.595, P=0.026; 2011: F_2,18.030_=3.779, P=0.043) and mature height (2010: F_2,125_=25.749, P<0.001; 2011: F_2,136_=18.586, P<0.001) showed similar regional results to the full dataset, with Northeast plants shortest on average.

When the two highest-latitude populations (populations 14 and 18) were removed from the datasets, region no longer significantly contributed to variation in transplant height and flowering height in either year (with the exception of transplant height in 2010), although the posthoc tests reveal that the Northeast region remained significantly shorter at transplantation and flowering in both years (S4 and S5 Tables). In contrast, mature height remained significantly different between regions in both years (F_2,232_=22.366, P<0.001 in 2010; F_2,342_=24.946, P<0.001 in 2011), and all other modeled sources of variation also remained significant. Plant height over time provides an approximate measure of growth rate, and these data suggest that Northeastern plants grow more slowly (and mature at smaller stature) than plants from the other regions, and that the relative average mature height of Mississippi Valley and Plains individuals may depend on common garden location.

### Mature plant data

To estimate of the size of mature plants, three measurements were taken just before plants were harvested: height, branch number, and length of the longest branch. These measurements, along with dry above-ground biomass, were moderately positively correlated with each other (S6 Table); therefore, the data were analyzed together in a multivariate GLM analysis, as well as in univariate GLMs. In the multivariate GLM analyses of the full dataset, all of the modeled sources of variation significantly affected mature data in both years, with the exception of the covariate, transplant height, which was non-significant in 2010 (Table 3). Notably, region of origin significantly contributed to variation in multivariate mature data in both common gardens (2010: F_8,494_=12.425, P<0.001; 2011: F_8,730_=27.530, P<0.001).

In the univariate GLMs for the full dataset, all of the modeled sources of variation also had significant effects on mature branch number in both years, with the exception of transplant height in 2010 (Table 2). Posthoc tests showed that in 2010, Mississippi Valley plants had the most branches, followed by Plains plants, and Northeast plants had the fewest (P_1,2_ and P_2,3_<0.001, and P_1,3_=0.011), while in 2011, Mississippi Valley plants had significantly more branches than the other two regions (P_1,2_<0.001 and P_2,3_=0.009; S2 Fig). Similarly, dry above-ground biomass was significantly affected by all of the modeled sources of variation except for the covariate (transplant height) in both years (Table 2); region of origin was significant in 2010 (F_2,249_=13.228, P<0.001) and in 2011 (F_2,367_=29.871, P<0.001). In 2010, Mississippi Valley plants had more biomass on average than plants from the other two regions (posthoc tests: P_1,2_=0.001 and P_2,3_<0.001; Fig 4A); in 2011, Mississippi Valley and Plains plants had more biomass on average than Northeast plants (posthoc tests: P_1,3_ and P_2,3_<0.001; Fig 4B). The length of the longest mature branch was not significantly impacted by region (or anything but block) in 2010, whereas in 2011, the variation in this measure was significantly affected by everything but the covariate transplant height (Table 2), and Mississippi Valley and Plains plants had significantly longer longest branches than Northeast plants (posthoc tests: P_1,3_ and P_2,3_<0.001). Just as with height data, the effect of region was not significantly different in different blocks (no block by region interactions) for mature plant data, so block-by-block results were not examined.

In both years, female-only dry biomass (2010: F_2,125_=11.002, P<0.001; 2011: F_2,136_=6.507, P=0.002) showed similar regional results to the full dataset, with Northeast plants having the least biomass on average. Branch number at maturity exhibited slightly different patterns when male plants were excluded: block had no significant impact on variation in this measure, in contrast to the full dataset, and the covariate transplant height (which was barely significant in 2011) was not significant in either year with just female branch number data (again, probably due to smaller sample sizes). In addition, the regional branch number means for females alone exhibited altered magnitude of differences (although their relative relationships remain the same), leading to different pairwise significance values in the posthoc tests (S3 Table). The length of the longest branch had fewer factors significantly impacting its variation in 2011 when only female data is analyzed: in contrast to the full dataset (for which only transplant height had no significant effect on branch length), there was no significant effect of region, population nested within region, or transplant height, and none of the regions were different in the posthoc tests.

When data from the high latitude populations 14 and 18 were omitted, mature branch number and length of the longest mature branch still exhibited the same overall patterns with respect to factors influencing their variation: the exceptions (transplant height was no longer significant in 2011 for branch number, and region was no longer significant in 2011 for branch length) can be attributed largely to reduced sample size (S4 Table). The regional means had the same relative magnitudes for both branch number and branch length in 2010, and for branch length in 2011, although in 2011, the omission of these populations caused the average Northeast branch number to be statistically indistinguishable from that of the Mississippi Valley (in contrast to the full dataset, when the Northeast more closely resembled the Plains) (S5 Table).

Without data from populations 14 and 18, dry biomass in 2010 was still significantly different between regions (F_2,232_=8.516, P<0.001; driven by the larger biomass values of the Mississippi Valley), but biomass in 2011 was not significantly different between regions overall or between any two regions (although the mean biomass of the Northeast was smaller than that of the Plains or the Mississippi Valley) (S4 and S5 Tables). Population-level analysis showed that in 2010, population 14 (Northeast) had the lowest average biomass, significantly lower than many of the other populations (1 (Plains), 9, 10, 11, 12 (Mississippi Valley), and 15 (Northeast)) (S3A Fig). In 2011, populations 14 and 18 (Northeast) had by far the lowest biomass (significantly lower than all other populations) (S3B Fig). Taken together with the previously reported results for mature height, these results suggest that Northeastern plants are generally smaller overall at maturity in height and biomass (and less so for branch number and length of the longest branch, particularly with the omission of the highest-latitude populations), but that the relative average size of Mississippi Valley and Plains individuals may depend on common garden location.

### Days to flowering

#### Regional variation

Kruskal-Wallis tests of the ordinal data “days to flower,” the number of days from planting to flowering, showed that Northeast plants took significantly fewer days to flower in both years (2010: df=2, χ^2^=12.237, P=0.002; Northeast plants taking an average of 6.38 days fewer to flower than the other two regions averaged together; 2011: df=2, χ^2^=11.542, P=0.003; Northeast plants taking an average of 4.34 days fewer to flower than Mississippi Valley plants) (Table 2; S1 Table, S4 Fig). Overall, 2010 plants took more days to flower than 2011 plants on average (9.17 days more); this is presumably because waterhemp planting in 2011 took place later in the year (June 10, vs. May 19 in 2010). With female data alone, days to flowering was almost significantly different between regions in the 2010 experiment (df=2, χ^2^=6.004, P=0.050), and Northeast plants flowered in significantly fewer days (6.34 fewer days) than Mississippi Valley plants, but neither region was significantly different from Plains plants (in contrast to the full dataset) (S2 and S3 Tables). In 2011, days to flowering was significantly different between regions (df=2, χ^2^=9.295, P=0.010), but Plains plants flowered in significantly fewer days than Mississippi Valley plants (while neither was different from Northeast plants), swapping the pattern exhibited by Plains vs. Northeast regions in the full dataset.

#### Latitudinal variation

Days to flowering results by region were correlated with latitude of origin in 2010, with four out of six Northeast populations coming from relatively high latitude sites. When two of these high-latitude populations were replaced with lower-latitude Northeast populations between 2010 and 2011, this region still took significantly fewer days to start flowering. However, when data from the two remaining highest latitude populations (populations 14 and 18, found at ~43°N) were removed from the analyses, days to flowering differences between regions become non-significant for both years (S4 and S5 Tables). Days to flowering showed a significant association with latitude of population of origin in both years (Kruskal-Wallis test of independent samples for average days to flowering by latitude: P<0.001 in both years), with plants from higher-latitude populations flowering in significantly fewer days (Fig 5; S5 Fig). This suggests that Northeast plants respond differently to photoperiod or other phenological cues than do Plains or Mississippi Valley plants, but that this difference may be solely attributable to differences in latitude of origin.

## Discussion

We set out to explore potential variation in agricultural adaptation in a Midwestern native plant species, waterhemp (*Amaranthus tuberculatus* s.l.). We conducted two common garden experiments in different geographical locations in 2010 and 2011, inside and outside of the range of agriculturally weedy waterhemp, to test the hypothesis that waterhemp plants from the Mississippi Valley region have higher fitness in agricultural environments than do plants from less heavily infested regions (the Plains and Northeast). We discovered that different components of fitness (days to flowering, height over time, and measures of mature plant size) displayed disparate patterns in our results, influenced by region of origin, latitude of origin, and year/location of experimentation (Table 2; Figs 2 to 5). Specifically, height and mature plant size showed patterns suggesting agricultural adaptation by region, whereas days to flowering showed a strong effect of genetic adaptation of individual populations to their latitude of origin. These results suggest that waterhemp has the capacity to adapt to different environments across its range, and that modern agricultural fields are likely a key environment to which it has recently adapted. However, some of our results could also potentially be explained by alternative scenarios, such as agricultural “preadaptation” in part of waterhemp’s geographical range. Below we discuss the implications and limitations of our findings.

### Regional and latitudinal variation in agricultural fitness

The data collected for the height and size/biomass components of fitness lend some support to the hypothesis of agricultural adaptation in Mississippi Valley populations. Height over time and dry above-ground biomass showed largely congruent patterns, with Northeast plants growing more slowly and maturing at smaller stature and weight on average than plants from the other two regions in both years. The height and biomass of Mississippi Valley plants relative to Plains plants was dependent on the year/location (greater in 2010, but not in 2011). There was also a significant impact of sex on these vegetative growth traits, with female plants always taller and heavier (which is frequently the case for dioecious herbaceous plants; [56]). When only data from female plants were analyzed, the height and biomass results showed the same patterns as for the full dataset, with most differences in significance attributable to smaller sample sizes when only female data are included; thus, female and male plants responded in a similar fashion to the experimental conditions. Mature branch number and length of the longest mature branch show results that do not consistently support the hypothesis, when examining the results between years (in 2011, Plains plants had the fewest branches), between sexes (male plants are driving the significant effect of region on branch length in the 2011 GLM), and with the omission of the data from the highest-latitude populations (in 2011, the remaining Northeastern plants have the same average number of branches as the Mississippi Valley plants).

On average, Northeast plants reached a smaller maximum height and above-ground biomass than plants from the other two regions; however, the patterns for biomass in 2011 were more complicated. The highest-latitude populations from the Northeast (Populations 14 and 18) had low biomass in both years, and they were largely responsible for the significant biomass differences observed between regions in 2011. The 2011 biomass results could have been driven by the shorter growing season due to earlier flowering in the highest-latitude Northeast populations, combined with the fact that the Ohio common garden was initiated a full month later in the growing season than the 2010 Missouri common garden. However, one caveat to this conclusion is that the association between latitude and biomass was only observed for the highest-latitude populations in the Northeast region; two of the populations from the Mississippi Valley (7 and 8) were found at nearly the same latitude as these high-latitude Northeast populations (Table 1), and yet the height and biomass of plants from these locations were largely similar to other Mississippi Valley populations (S1 and S3 Figs). The fact that the latitudinal influence on vegetative growth-related fitness traits appears to be specific to the Northeast region lends credence to the idea that days to flowering alone is not the sole driver of the 2011 biomass results.

From the generally similar height and biomass results derived from the analysis of females alone compared to the full data set, we can conclude that harvesting male plants earlier in 2011 than in 2010 did not significantly change the patterns in these data. Additionally, the two agricultural populations that were included affected the results to a very minor extent, and actually made the results less significantly different between regions, rather than more different (S7 and S8 Tables). However, the later planting date and randomly-placed soybeans in the Ohio plots undoubtedly did contribute to the difference in results between years, as plants in the 2011 common garden were on average taller, heavier, and flowered in fewer days than 2010 plants, probably due to timing of soybean planting leading to less crop competition with the weeds. Indeed, region-based differences in plant size overall were reduced in 2011 relative to 2010, probably also due to later initiation of the experiment (S1 Table). Therefore, we cannot confidently conclude that the reduced difference between the Mississippi Valley and Plains measurements for most datasets in the Ohio common garden (relative to Missouri) was attributable to a loss of “home advantage” from local adaptation of the Mississippi Valley plants to the Missouri common garden conditions. The only way to accurately evaluate the impact of these factors would be to perform more years of common garden studies to control for inevitable climate and pest variation between any two years of outdoor research.

In our experiments, days to flowering (a measure of flowering time in our experiment) was driven strongly by latitude of origin in both 2010 and 2011 (Fig 5). Northeast plants took 4 to 7 fewer days to flower on average than plants from the other two regions; however, this relationship was weakened when Northeast plants from two high-latitude populations (over 41.5°N) were replaced by lower-latitude populations in the experiment (in 2011 vs. 2010), and disappeared altogether when the remaining high-latitude Northeast populations were removed from the data analysis. *Amaranthus tuberculatus* s.l. is a short-day plant [57], although photoperiod is only one of the factors controlling flowering time (e.g., plants will flower when very small if pot-bound [K. Waselkov, pers. obs.]). In the course of our seed collections in the field, we observed that crop field waterhemp populations typically flower earlier than nearby riverbank populations in the agricultural waterhemp regions, despite the near-certainty of high gene flow between these populations; this suggests that waterhemp flowering phenology responds plastically to agricultural practices and riverbank inundation. Flowering is the beginning of senescence for waterhemp individuals, particularly for males (which took an average of 9-10 days fewer to flower than females in our study, a pattern consistent with previous observations [58]); thus, earlier flowering limits the size that a waterhemp plant can attain during the growing season. Shifting relationships between regions in days to flowering when males are excluded suggests that males and females may truly exhibit different patterns in this variable.

Life history events such as flowering are phenotypically plastic traits under strong selection in crop fields, because a weed’s growing season is entirely bounded by crop planting and harvest [13, 59]. Many researchers have recognized the importance of phenology to establishment and competitive ability of agricultural weeds (i.e., [20, 60, 61]). Examination of life history traits such as flowering time, fecundity, and dormancy suggests that variation in agricultural practices can select for different life-history strategies in a single species, as observed in *Capsella bursa-pastoris* in the UK [62]. Our findings suggest that within the species *Amaranthus tuberculatus* s.l., there is significant genetic variation for the phenotypically plastic life history trait of days to flowering.

### Agricultural “preadaptation”

Overall, our results provide evidence that there are adaptive differences between waterhemp populations from different geographical regions. However, there is another potential explanation for the generally lower fitness of Northeast plants as agricultural weeds. Size of individual organisms across a species range is predicted to be variable, according to one ecological theory: sizes may be smaller in peripheral populations, relative to the center of the range, as ecological conditions become less suitable for the species [63], although evidence supporting this prediction is equivocal [64, 65]. This is related to the concept of “preadaptation” (from invasion biology and weed science), in which some species or populations of plants may have evolved the characteristics needed for successful invasion and competition in new habitats, before they are introduced to such environments, in their native range [66]. This is distinct from another evolutionary definition of preadaptation, as an alternative term for exaptation (which involves a change in trait function); to distinguish the invasion biology concept from exaptation, some authors prefer the terminology “prior adaptation” rather than “preadaptation” [67].

In 2010, we conducted a simultaneous waterhemp population genetic study with 10 microsatellite markers, with sampling from across the species range (and some of the same populations included in the present study). This research revealed two genetic subpopulations within waterhemp, roughly divided geographically by the Mississippi River into an “eastern” and a “western” group, but with some populations east of the Mississippi (in Illinois, Indiana, and Ohio) in areas with agricultural waterhemp infestations, showing genetic affinity to the western group [52]. The western genetic subpopulation chiefly corresponded taxonomically to *A*. *tuberculatus* var. *rudis*, and the eastern genetic subpopulation corresponded to *A*. *tuberculatus* var. *tuberculatus*. Although not conclusive, the combination of our common garden fitness data and genetic results strongly suggests that Mississippi Valley and/or Plains populations were predisposed, or preadapted, to invade Mississippi Valley agricultural environments when the opportunity presented itself in the 20^th^ century, rather than requiring genetic changes to become successful in these new habitats. The genetic similarity between sampled populations from the Mississippi Valley and Plains regions (despite the different levels of agricultural infestation in these regions) and the dissimilarity from the sampled Northeast populations indicate that the “western” genetic variety may have already possessed the qualities necessary to compete with crops [52]. However, from our experiments, we cannot pinpoint exactly which morphological or physiological traits lead to higher fitness (as measured grossly by height and biomass) in the Mississippi Valley/Plains plants. This would require multifactorial common gardens or controlled greenhouse experiments.

The question of agricultural preadaptation has seldom been addressed, because few weeds have invaded agricultural environments recently enough to permit examination of “before and after” populations. Waterhemp is unusual in that the approximate time and location of its agricultural invasion are known [29]. In invasion biology, there is much interest in predicting invasiveness based on particular morphological, physiological, or life history traits [68, 69], and several researchers have taken advantage of knowing the details of recent invasions to compare these traits in conspecific invasive and native populations (e.g., [70–72]). In general, these studies have shown greater fitness of the invasive populations, suggesting genetic adaptation rather than preadaptation. More recently, invasion biologists have begun to take advantage of advances in genetics, by combining population structure analyses and quantitative genetic and/or experimental studies of adaptation across the full native and introduced ranges of an invasive species. The design of these studies can shed light on whether populations from a particular geographic area or habitat within the native range are locally adapted in a way that facilitated establishment of invasive populations in a similar habitat after introduction [73–75]. A particular mechanism hypothesized to lead to preadaptation called “anthropogenically induced adaptation to invade” (AIAI) may be relevant in the case of waterhemp: in this scenario, local adaptation to human-altered habitats (such as crop fields) in the native range could: a) predispose the populations to be successful in similar environments in the introduced range, and; b) make the transport of their propagules between the native and introduced ranges more likely (for instance, by farm machinery that is being moved long distances) [57, 67]. Ultimately, it may be impossible to rigorously test the hypothesis of preadaptation in *A*. *tuberculatus* var. *rudis*, as it is native to part of the same region where it is an agricultural field invader, and gene flow is assumed to have been continuous between sympatric agricultural and riparian environments since the beginning of its colonization of agroecosystems (circa 1850) [29].

### Suggestions for further work

Waterhemp exhibits discontinuous germination, also known as an extended emergence pattern, in which seeds continue to germinate throughout the growing season, limiting the potential options for weed control [32, 46, 58]. Our experiments were not designed to measure seed dormancy and germination traits, which several previous publications have shown to vary among waterhemp populations and among tillage systems [33, 76, 77]. A particularly interesting study showed, albeit with single individuals, that an Ohio riverbank plant had much lower seed dormancy than two Iowa agricultural plants [78]. The present study minimized the impact of seed dormancy differences on fitness by stratifying all the seeds, which made germination percentages more similar between waterhemp populations with different dormancy levels in Leon et al.’s [78] study. Future common garden experiments with natural waterhemp populations should aim to incorporate seed dormancy characteristics, as these traits have a large impact on fitness in waterhemp and other agricultural weeds [79–81].

Finally, common gardens are the standard type of experiment for studies of local adaptation and intraspecific trait variation [82, 83]. Reciprocal transplant experiments are even more sophisticated, as they measure the performance of plants in each other’s native environments [84]. Therefore, the ideal waterhemp garden experiment would be reciprocal transplants of waterhemp from the agriculturally invaded and uninvaded ranges into both soybean plots and riverbank plots. Unfortunately, problems with extensive riverbank flooding in 2010 prevented transplantation of waterhemp into riverbank plots in Missouri (as originally planned). However, for future studies, paired, replicated riverbank and crop field plots, in several sites inside and outside the range of agricultural waterhemp and at different latitudes, would be the most comprehensive way to study fitness and local/agricultural adaptation in this system. These experiments would shed further light on whether the small size and early flowering of Northeast waterhemp is adaptive in the environments where it naturally occurs. They could also be designed to test for fitness tradeoffs resulting from herbicide resistance, which have seldom been documented in *Amaranthus* species for resistance to herbicides other than atrazine [85–89].

## Conclusions

We found evidence that *Amaranthus tuberculatus* s.l. from the geographical region where the species is highly agriculturally invasive may be better adapted to crop field environments than plants from populations outside this region. Latitude of origin also has a significant impact on agricultural fitness of waterhemp populations. These results have implications for the evolution of new native agricultural weeds, particularly the evidence for preadaptation of a subset of *A*. *tuberculatus* s.l. to crop fields: many species in naturally disturbed environments like riverbanks may already have traits that would confer high fitness in agricultural environments, and their invasion could be precipitated by changes in management practices (such as conservation tillage and reliance on herbicide, in the case of waterhemp). Studying similar agricultural weeds that have invaded crop fields in their native range could also reveal patterns of intraspecific genetic variation that correlate with agricultural preadaptation, or adaptation since agricultural incursion. Our results are the latest in a growing body of evidence that evolutionary factors, such as population structure and adaptive genetic variation, are important in shaping invasiveness in agricultural weeds, as well as invaders of more natural ecosystems.

## Supporting information

S1 Fig Box plots of mature height (cm) of plants over time, by population of origin. A = 2010, B = 2011. Letters next to box plots represent groups that are significantly different (different letters) or are not significantly different (same letters) as determined by post-hoc tests. Circles represent outliers (cases with values between 1.5 and 3 times the interquartile range). Asterisks represent extreme outliers (cases with values greater than 3 times the interquartile range). Blue = Plains populations, red = Mississippi Valley populations, gold = Northeast populations.
(TIFF)

S2 Fig Box plots of square-root-transformed mature branch number, by region of origin. A = 2010, B = 2011. Letters next to box plots represent groups that are significantly different (different letters) or are not significantly different (same letters) as determined by post-hoc tests. Circles represent outliers (cases with values between 1.5 and 3 times the interquartile range).
(TIFF)

S3 Fig Box plots of log_10_-transformed dry above-ground biomass, by population of origin. A = 2010, B = 2011. Letters next to box plots represent groups that are significantly different (different letters) or are not significantly different (same letters) as determined by post-hoc tests. Circles represent outliers (cases with values between 1.5 and 3 times the interquartile range). Blue = Plains populations, red = Mississippi Valley populations, gold = Northeast populations.
(TIFF)

S4 Fig Cumulative percentage of individuals flowering in each time interval, measured in days from planting to flowering, by region of origin. A = 2010, B = 2011.
(TIFF)

S5 Fig Box plots of days to flowering by latitude of origin (i.e., population, rearranged by increasing latitude, treated as a categorical variable). A = 2010, B = 2011. Letters next to box plots represent groups that are significantly different (different letters) or are not significantly different (same letters) as determined by post-hoc tests. Circles represent outliers (cases with values between 1.5 and 3 times the interquartile range). Asterisks represent extreme outliers (cases with values greater than 3 times the interquartile range). Blue = Plains populations, red = Mississippi Valley populations, gold = Northeast populations. 2010 population number order (left to right): 1, 5, 2, 11, 10, 15, 9, 16, 6, 4, 12, 3, 13, 8, 7, 17, 14, 18. 2011 population number order (left to right): 1, 5, 2, 11, 10, 15, 9, 16, 13, 6, 17, 4, 12, 3, 8, 7, 14, 18.
(TIFF)

S1 Table Mean values, standard deviations, and samples sizes for transplant height, flowering height, mature height, mature branch number, length of longest mature branch, dry above-ground biomass, and days to flowering by region (full data set). SD = standard deviation, N = sample size, with number of female samples in parentheses. Letters in the “post-hoc test results” row represent groups that are significantly different (different letters) or are not significantly different (same letters) with alpha = 0.05, as determined by post-hoc tests.
(DOCX)

S2 Table Results from general linear models (GLM) or nonparametric Kruskal-Wallis tests of the effect of fixed and random factors on transplant height, flowering height, mature height, mature branch number, length of longest mature branch, dry above-ground biomass, and days to flowering (just female plants). Significant values at alpha = 0.05 are bold.
(DOCX)

S3 Table Mean values, standard deviations, and samples sizes for transplant height, flowering height, mature height, mature branch number, length of longest mature branch, dry above-ground biomass, and days to flowering by region (just female plants). SD = standard deviation, N = sample size. Letters in the “post-hoc test results” row represent groups that are significantly different (different letters) or are not significantly different (same letters) with alpha = 0.05, as determined by post-hoc tests.
(DOCX)

S4 Table Results from general linear models (GLM) or nonparametric Kruskal-Wallis tests of the effect of fixed and random factors on transplant height, flowering height, mature height, mature branch number, length of longest mature branch, dry above-ground biomass, and days to flowering (populations 14 and 18 omitted). The Northeast region has had data from populations 14 and 18 omitted. Significant values at alpha = 0.05 are bold.
(DOCX)

S5 Table Mean values, standard deviations, and samples sizes for transplant height, flowering height, mature height, mature branch number, length of longest mature branch, dry above-ground biomass, and days to flowering by region (populations 14 and 18 omitted). SD = standard deviation, N = sample size. The Northeast region has had populations 14 and 18 omitted. Letters in the “post-hoc test results” row represent groups that are significantly different (different letters) or are not significantly different (same letters) with alpha = 0.05, as determined by post-hoc tests.
(DOCX)

S6 Table Pearson correlation matrices for mature plant data, 2010 and 2011 common gardens. (DOCX)

S7 Table Results from general linear models (GLM) or nonparametric Kruskal-Wallis tests of the effect of fixed and random factors on transplant height, flowering height, mature height, mature branch number, length of longest mature branch, dry above-ground biomass, and days to flowering (populations 7 and 12 omitted). The Mississippi Valley region has had data from populations 7 and 12 omitted. Significant values at alpha = 0.05 are bold.
(DOCX)

S8 Table Mean values, standard deviations, and samples sizes for transplant height, flowering height, mature height, mature branch number, length of longest mature branch, dry above-ground biomass, and days to flowering by region (populations 7 and 12 omitted). SD = standard deviation, N = sample size. The Mississippi Valley region has had populations 7 and 12 omitted. Letters in the “post-hoc test results” row represent groups that are significantly different (different letters) or are not significantly different (same letters) with alpha = 0.05, as determined by post-hoc tests.
(DOCX)

S9 Table Climatic data and soil type data for 2010 and 2011 common garden locations. Climatic data for the 2010 common garden (in Eureka, MO) is from the St. Louis International Airport; climatic data for the 2011 common garden (in Oxford, OH) is from the Hamilton-Butler County Regional Airport. Climatic data downloaded from the NOAA National Centers for Environmental Information’s Climate Data Online (http://ncdc.noaa.gov/cdo-web/); soil type data downloaded from the USDA Natural Resources Conservation Service’s Web Soil Survey (http://websoilsurvey.nrcs.usda.gov/app/).
(DOCX)

S1 Methods and Results (DOCX)
