# Supplementary figures and images for: Agricultural adaptation in the native North American weed waterhemp, Amaranthus tuberculatus (Amaranthaceae)
Source: PLoS One. 2020 Sep 24;15(9):e0238861. doi: 10.1371/journal.pone.0238861 (PMC7514059; doi:10.1371/journal.pone.0238861)

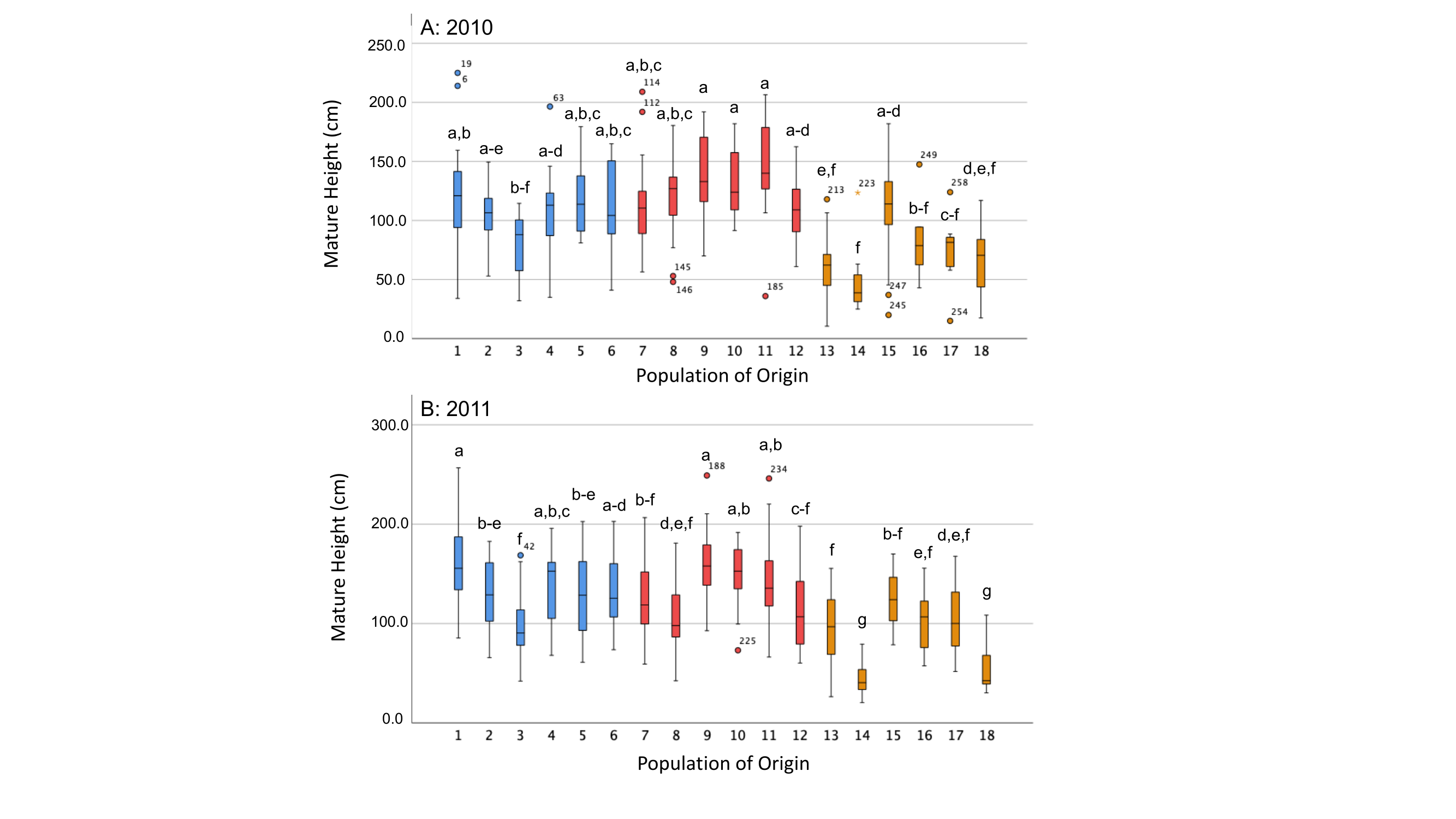

Supplement: S1 Fig — A = 2010, B = 2011. Letters next to box plots represent groups that are significantly different (different letters) or are not significantly different (same letters) as determined by post-hoc tests. Circles represent outliers (cases with values between 1.5 and 3 times the interquartile range). Asterisks represent extreme outliers (cases with values greater than 3 times the interquartile range). Blue = Plains populations, red = Mississippi Valley populations, gold = Northeast populations. (TIFF) [file pone.0238861.s001.tiff]

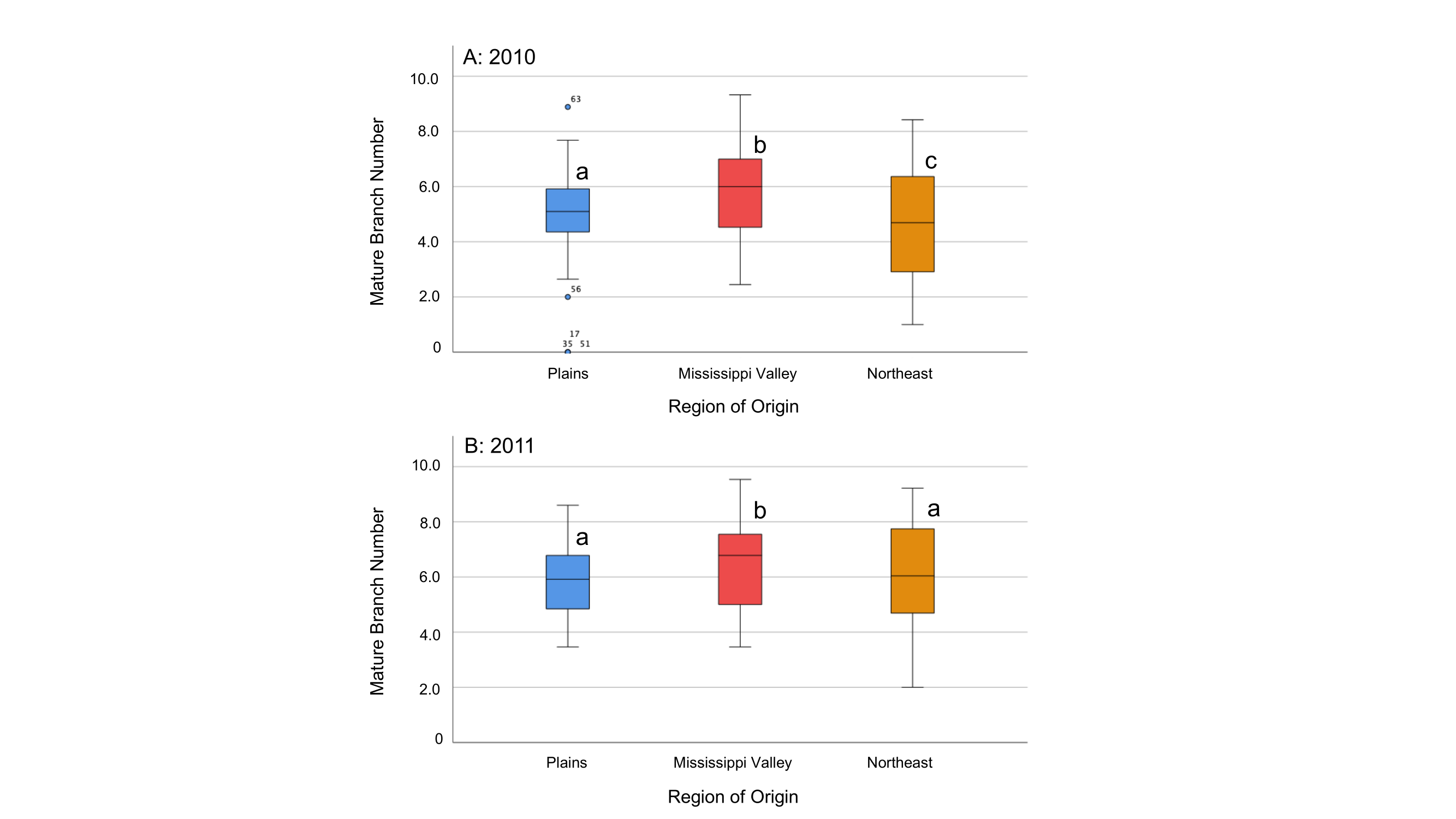

Supplement: S2 Fig — A = 2010, B = 2011. Letters next to box plots represent groups that are significantly different (different letters) or are not significantly different (same letters) as determined by post-hoc tests. Circles represent outliers (cases with values between 1.5 and 3 times the interquartile range). (TIFF) [file pone.0238861.s002.tiff]

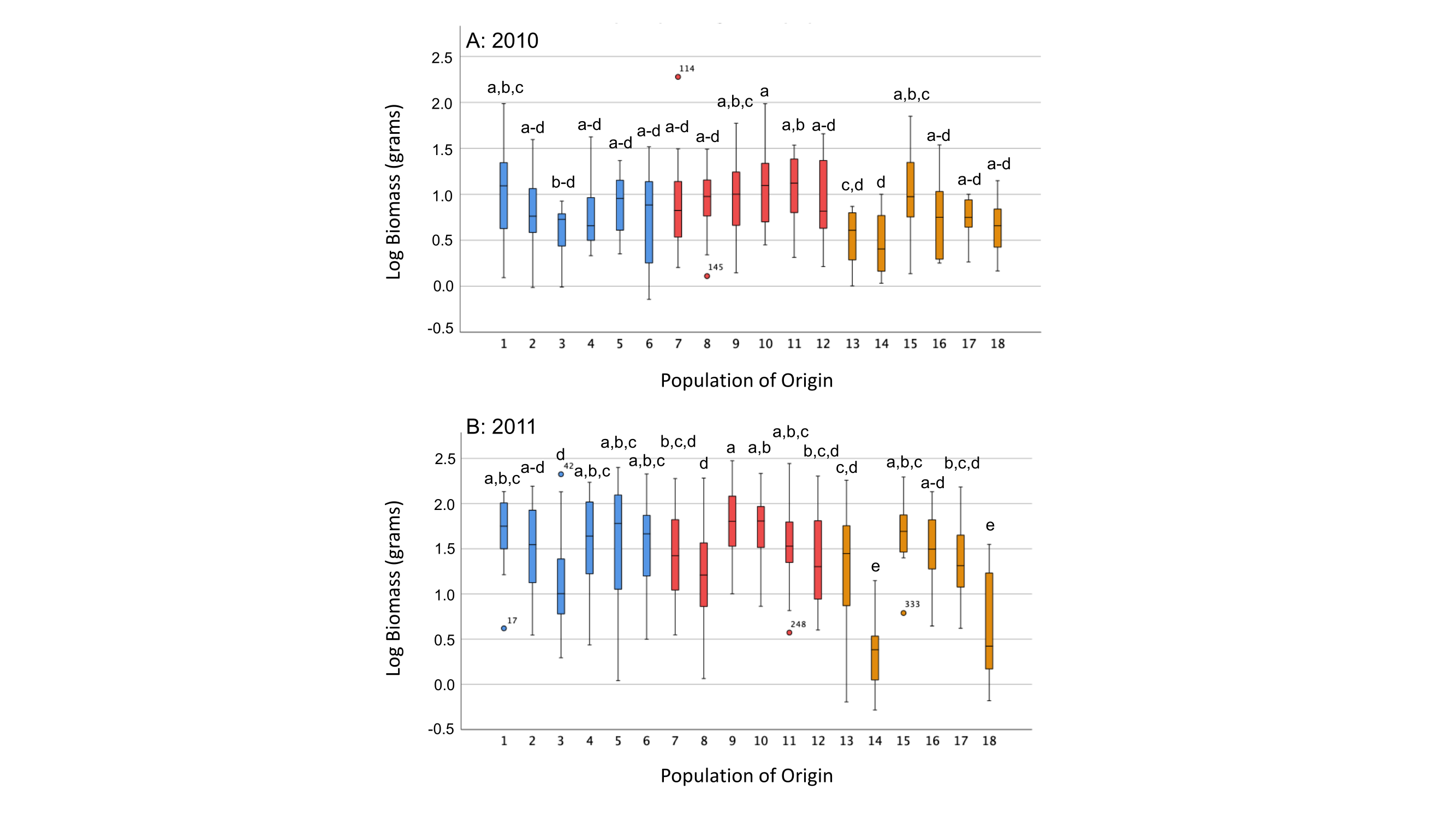

Supplement: S3 Fig — A = 2010, B = 2011. Letters next to box plots represent groups that are significantly different (different letters) or are not significantly different (same letters) as determined by post-hoc tests. Circles represent outliers (cases with values between 1.5 and 3 times the interquartile range). Blue = Plains populations, red = Mississippi Valley populations, gold = Northeast populations. (TIFF) [file pone.0238861.s003.tiff]

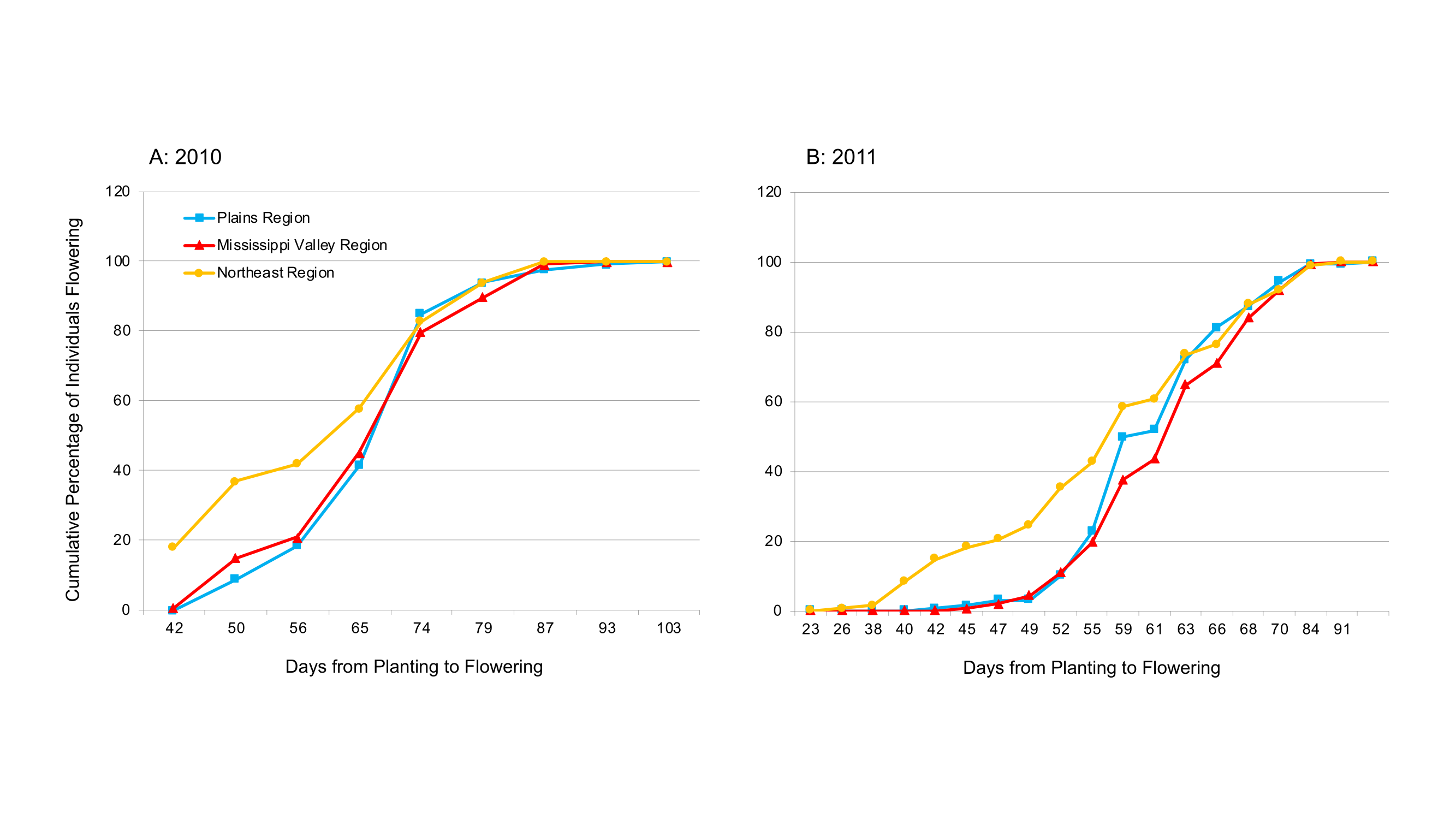

Supplement: S4 Fig — A = 2010, B = 2011. (TIFF) [file pone.0238861.s004.tiff]

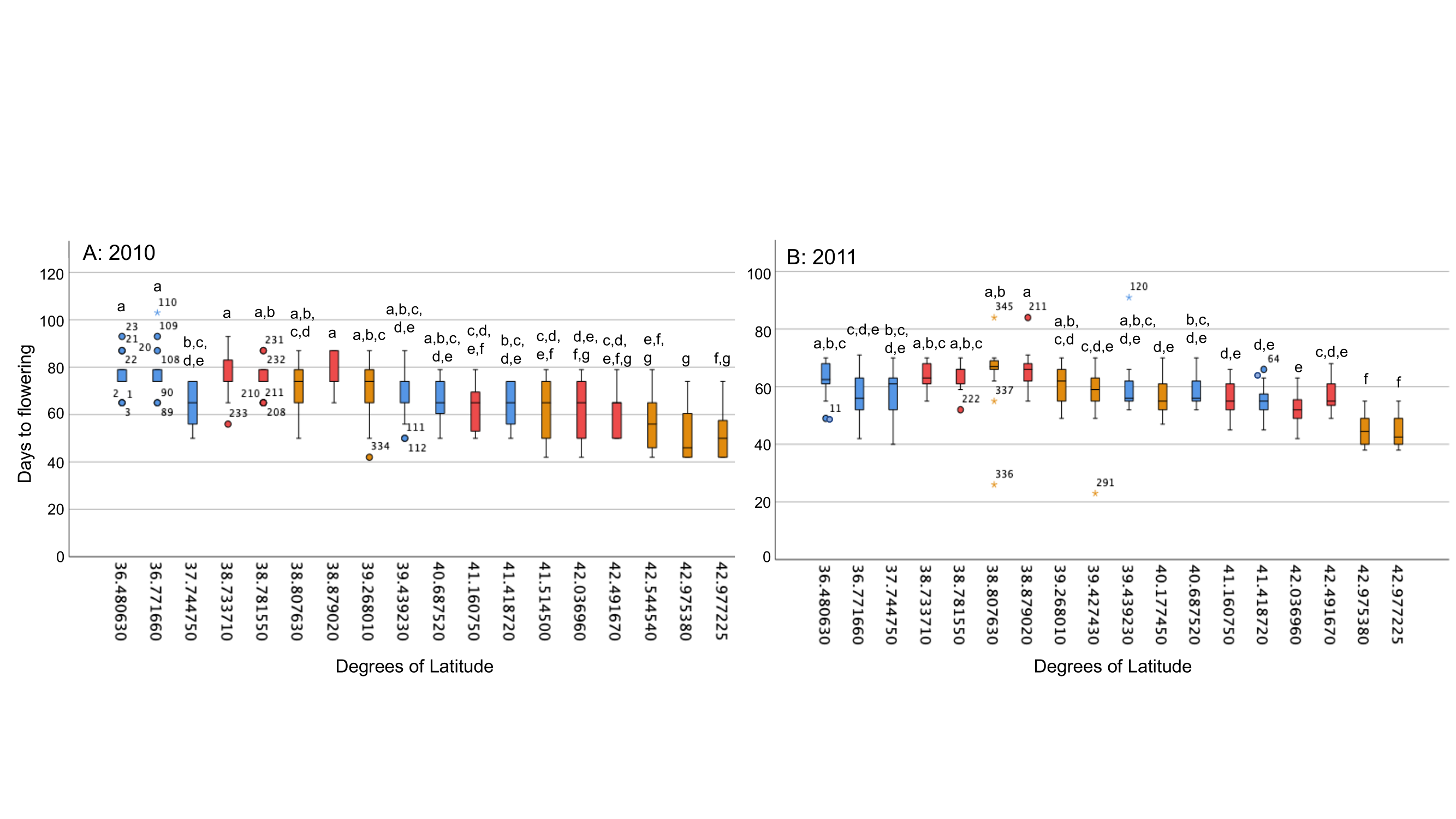

Supplement: S5 Fig — A = 2010, B = 2011. Letters next to box plots represent groups that are significantly different (different letters) or are not significantly different (same letters) as determined by post-hoc tests. Circles represent outliers (cases with values between 1.5 and 3 times the interquartile range). Asterisks represent extreme outliers (cases with values greater than 3 times the interquartile range). Blue = Plains populations, red = Mississippi Valley populations, gold = Northeast populations. 2010 population number order (left to right): 1, 5, 2, 11, 10, 15, 9, 16, 6, 4, 12, 3, 13, 8, 7, 17, 14, 18. 2011 population number order (left to right): 1, 5, 2, 11, 10, 15, 9, 16, 13, 6, 17, 4, 12, 3, 8, 7, 14, 18. (TIFF) [file pone.0238861.s005.tiff]
