# Supplementary material for: Agricultural adaptation in the native North American weed waterhemp, Amaranthus tuberculatus (Amaranthaceae)
Source: PLoS One. 2020 Sep 24;15(9):e0238861. doi: 10.1371/journal.pone.0238861 (PMC7514059; doi:10.1371/journal.pone.0238861)
Supplement: S1 Table — SD = standard deviation, N = sample size, with number of female samples in parentheses. Letters in the “post-hoc test results” row represent groups that are significantly different (different letters) or are not significantly different (same letters) with alpha = 0.05, as determined by post-hoc tests. (DOCX) [file pone.0238861.s006.docx]

**S1 Table.** **Mean values, standard deviations, and samples sizes for transplant height, flowering height, mature height, mature branch number, length of longest mature branch, dry above-ground biomass, and days to flowering by region (full data set).** SD = standard deviation, N = sample size, with number of female samples in parentheses. Letters in the “post-hoc test results” row represent groups that are significantly different (different letters) or are not significantly different (same letters) with alpha = 0.05, as determined by post-hoc tests.

|  | 2010 | |  |  |  | 2011 |  |  |
| --- | --- | --- | --- | --- | --- | --- | --- | --- |
| Transplant Height† | Plains | | Mississippi Valley | Northeast |  | Plains | Mississippi Valley | Northeast |
| Mean (SD) | 2.080 (0.313) | | 2.084 (0.288) | 1.856 (0.302) |  | 9.824 (3.449) | 9.980 (2.684) | 7.925 (2.337) |
| N (N female) | 110 (59) | | 103 (54) | 66 (36) |  | 137 (62) | 153 (57) | 111 (39) |
| Post-hoc test results | A | | A | B |  | A | A | B |
|  | 2010 | |  |  |  | 2011 |  |  |
| Flowering Height | Plains | | Mississippi Valley | Northeast |  | Plains | Mississippi Valley | Northeast |
| Mean (SD) | 61.273 (27.064) | | 64.338 (36.117) | 37.324 (32.516) |  | 109.794 (31.406) | 115.985 (31.508) | 77.448 (29.327) |
| N (N female) | 110 (59) | | 103 (54) | 66 (36) |  | 137 (62) | 153 (57) | 111 (39) |
| Post-hoc test results | A | | A | B |  | A | A | B |
|  |  | |  |  |  |  |  |  |
| Mature Height | Plains | | Mississippi Valley | Northeast |  | Plains | Mississippi Valley | Northeast |
| Mean (SD) | 108.353 (34.468) | | 124.965 (37.954) | 78.405 (40.142) |  | 130.221 (39.737) | 132.784 (41.187) | 90.453 (39.170) |
| N (N female) | 109 (59) | | 99 (52) | 63 (35) |  | 135 (62) | 148 (56) | 106 (39) |
| Post-hoc test results | A | | B | C |  | A | A | B |
|  |  | |  |  |  |  |  |  |
| Mature Branch Number* | Plains | | Mississippi Valley | Northeast |  | Plains | Mississippi Valley | Northeast |
| Mean (SD) | 5.071 (1.423) | | 5.768 (1.658) | 4.630 (1.962) |  | 5.919 (1.281) | 6.421 (1.426) | 6.127 (1.710) |
| N (N female) | 109 (59) | | 99 (52) | 63 (35) |  | 135 (62) | 148 (56) | 106 (39) |
| Post-hoc test results | A | | B | C |  | A | B | A |
|  |  | |  |  |  |  |  |  |
| Length of Longest Mature Branch* | Plains | | Mississippi Valley | Northeast |  | Plains | Mississippi Valley | Northeast |
| Mean (SD) | 6.149 (2.324) | | 6.632 (2.389) | 6.504 (2.289) |  | 8.149 (2.325) | 8.249 (2.164) | 6.968 (2.566) |
| N (N female) | 109 (59) | | 99 (52) | 63 (35) |  | 135 (62) | 148 (56) | 106 (39) |
| Post-hoc test results | A | | A | A |  | A | A | B |
| Dry Above-ground Biomass^¶^ | Plains | | Mississippi Valley | Northeast |  | Plains | Mississippi Valley | Northeast |
| Mean (SD) | 0.807 (0.423) | | 0.976 (0.438) | 0.725 (0.441) |  | 1.499 (0.528) | 1.511 (0.504) | 1.209 (0.649) |
| N (N female) | 109 (59) | | 99 (52) | 63 (35) |  | 135 (62) | 148 (56) | 106 (39) |
| Post-hoc test results | A | | B | A |  | A | A | B |
|  |  | |  |  |  |  |  |  |
| Days to Flowering | Plains | | Mississippi Valley | Northeast |  | Plains | Mississippi Valley | Northeast |
| Mean (SD) | 69.490 (10.073) | | 68.940 (11.175) | 62.830 (14.494) |  | 58.420 (6.967) | 59.840 (7.008) | 55.500 (10.547) |
| N (N female) | 135 (69) | | 139 (66) | 101 (60) |  | 137 (62) | 153 (57) | 111 (39) |
| Post-hoc test results | A | | A | B |  | A | A/B | A/C |
| †square-root transformed data in 2010  *square-root transformed data  ^¶^log transformed data | |  |  |  |  |  |  |  |
