# Supplementary material for: Agricultural adaptation in the native North American weed waterhemp, Amaranthus tuberculatus (Amaranthaceae)
Source: PLoS One. 2020 Sep 24;15(9):e0238861. doi: 10.1371/journal.pone.0238861 (PMC7514059; doi:10.1371/journal.pone.0238861)
Supplement: S2 Table — Significant values at alpha = 0.05 are bold. (DOCX) [file pone.0238861.s007.docx]

**S2 Table.** **Results from general linear models (GLM) or nonparametric Kruskal-Wallis tests of the effect of fixed and random factors on transplant height, flowering height, mature height, mature branch number, length of longest mature branch, dry above-ground biomass, and days to flowering (just female plants).** Significant values at alpha = 0.05 are bold.

| GLM | 2010 Transplant Height^b^ | |  | | |  | 2011 Transplant Height |  |  |  |
| --- | --- | --- | --- | --- | --- | --- | --- | --- | --- | --- |
| Factor | df (Hypothesis, Error) | | F ratio | | | P-value | df (Hypothesis, Error) | F ratio | P-value |  |
| Intercept | 1, 22.778 | | 5679.839 | | | **<0.001** | 1, 18.661 | 454.728 | **<0.001** |  |
| Region | 2, 20.437 | | 6.305 | | | **0.007** | 2, 17.173 | 3.213 | 0.065 |  |
| Population (Region) | 15, 131 | | 0.952 | | | 0.509 | 15, 140 | 4.013 | **<0.001** |  |
|  |  | |  | | |  |  |  |  |  |
| GLM | 2010 Flowering Height^a^ | |  | | |  | 2011 Flowering Height^a^ |  |  | |
| Factor | df (Hypothesis, Error) | | F ratio | | | P-value | df (Hypothesis, Error) | F ratio | P-value | |
| Intercept | 1, 79.635 | | 31.479 | | | **<0.001** | 1, 55.768 | 87.751 | **<0.001** | |
| Region | 2, 16.557 | | 4.595 | | | **0.026** | 2, 18.030 | 3.779 | **0.043** | |
| Population (Region) | 15,128 | | 4.209 | | | **0.001** | 15, 137 | 3.380 | **<0.001** | |
| Block | 2, 128 | | 7.196 | | | **<0.001** | 2, 137 | 3.246 | **0.042** | |
| Transplant Height | 1,128 | | 3.005 | | | 0.085 | 1, 137 | 3.588 | 0.060 | |
|  |  | |  | | |  |  |  |  | |
| GLM | 2010 Mature Height^a^ | |  | | |  | 2011 Mature Height^a^ |  |  | |
| Factor | df (Hypothesis, Error) | | F ratio | | | P-value | df (Hypothesis, Error) | F ratio | P-value | |
| Corrected Model | 20 | | 5.990 | | | **<0.001** | 20 | 5.371 | **<0.001** | |
| Intercept | 1 | | 30.644 | | | **<0.001** | 1 | 201.359 | **<0.001** | |
| Region | 2, 125 | | 25.749 | | | **<0.001** | 2, 136 | 18.586 | **<0.001** | |
| Population (Region) | 15, 125 | | 3.160 | | | **<0.001** | 15, 136 | 2.576 | **0.002** | |
| Block | 2, 125 | | 9.001 | | | **<0.001** | 2, 136 | 6.660 | **0.002** | |
| Transplant Height | 1, 125 | | 0.002 | | | 0.964 | 1, 136 | 1.054 | 0.307 | |
|  |  | |  | | |  |  |  |  | |
| GLM | 2010 Mature Branch Number^a,b^ | | |  | |  | 2011 Mature Branch Number^a,b^ |  |  | |
| Factor | df (Hypothesis, Error) | | F ratio | | | P-value | df (Hypothesis, Error) | F ratio | P-value | |
| Corrected Model | 20 | | 3.025 | | | **<0.001** | 20 | 3.299 | **<0.001** | |
| Intercept | 1 | | 81.407 | | | **<0.001** | 1 | 458.234 | **<0.001** | |
| Region | 2, 125 | | 11.241 | | | **<0.001** | 2, 136 | 10.010 | **<0.001** | |
| Population (Region) | 15, 125 | | 2.572 | | | **0.002** | 15, 136 | 2.680 | **0.001** | |
| Block | 2, 125 | | 2.859 | | | 0.061 | 2, 136 | 0.928 | 0.398 | |
| Transplant Height | 1, 125 | | 2.781 | | | 0.098 | 1, 136 | 0.073 | 0.788 | |
|  |  | |  | | |  |  |  |  | |
| GLM | 2010 Length of Longest Mature Branch^a,b^ | |  | | |  | 2011 Length of Longest Mature Branch^a,b^ |  |  | |
| Factor | df (Hypothesis, Error) | | F ratio | | | P-value | df (Hypothesis, Error) | F ratio | P-value | |
| Corrected Model | 20 | | 1.347 | | | 0.162 | 20 | 1.909 | **0.016** | |
| Intercept | 1 | | 10.267 | | | **0.002** | 1 | 246.539 | **<0.001** | |
| Region | 2, 125 | | 0.691 | | | 0.503 | 2, 136 | 1.564 | 0.213 | |
| Population (Region) | 15, 125 | | 0.642 | | | 0.835 | 15, 136 | 1.604 | 0.080 | |
| Block | 2, 125 | | 5.068 | | | **0.008** | 2, 136 | 4.821 | **0.009** | |
| Transplant Height | 1, 125 | | 1.504 | | | 0.222 | 1, 136 | 2.408 | 0.123 | |
|  |  | |  | | |  |  |  |  | |
| GLM | 2010 Dry Above-ground Biomass^a,c^ | | | |  |  | 2011 Dry Above-ground Biomass^a,c^ |  |  | |
| Factor | df (Hypothesis, Error) | | F ratio | | | P-value | df (Hypothesis, Error) | F ratio | P-value | |
| Corrected Model | 20 | | 3.561 | | | **<0.001** | 20 | 3.770 | **<0.001** | |
| Intercept | 1 | | 15.982 | | | **<0.001** | 1 | 302.116 | **<0.001** | |
| Region | 2, 125 | | 11.002 | | | **<0.001** | 2, 136 | 6.507 | **0.002** | |
| Population (Region) | 15, 125 | | 2.278 | | | **0.007** | 15, 136 | 2.461 | **0.003** | |
| Block | 2, 125 | | 7.584 | | | **0.001** | 2, 136 | 12.898 | **<0.001** | |
| Transplant Height | 1, 125 | | 0.115 | | | 0.735 | 1, 136 | 2.050 | 0.154 | |
|  |  | |  | | |  |  |  |  | |
| Kruskal-Wallis Test | 2010 Days to Flowering^d^ | |  | | |  | 2011 Days to Flowering^d^ |  |  | |
| Factor | df | | Chi-squared statistic | | | P-value | df | Chi-squared statistic | P-value | |
| Region | 2 | | 6.004 | | | 0.050 | 2 | 9.295 | **0.010** | |
| Significant values at alpha = 0.05 are bold. | |  |  | | |  |  |  |  | |
| ^a^With square-root transformed transplant height as a covariate (2010), or transplant height as a covariate (2011) | | | | | | | | | | |
| ^b^Square-root transformed data | | | | | | | | | | |
| ^c^Log transformed data  ^d^Categorical data |  | |  | | |  |  |  |  | |
