# Supplementary material for: Agricultural adaptation in the native North American weed waterhemp, Amaranthus tuberculatus (Amaranthaceae)
Source: PLoS One. 2020 Sep 24;15(9):e0238861. doi: 10.1371/journal.pone.0238861 (PMC7514059; doi:10.1371/journal.pone.0238861)
Supplement: S3 Table — SD = standard deviation, N = sample size. Letters in the “post-hoc test results” row represent groups that are significantly different (different letters) or are not significantly different (same letters) with alpha = 0.05, as determined by post-hoc tests. (DOCX) [file pone.0238861.s008.docx]

**S3 Table.** **Mean values, standard deviations, and samples sizes for transplant height, flowering height, mature height, mature branch number, length of longest mature branch, dry above-ground biomass, and days to flowering by region (just female plants).** SD = standard deviation, N = sample size. Letters in the “post-hoc test results” row represent groups that are significantly different (different letters) or are not significantly different (same letters) with alpha = 0.05, as determined by post-hoc tests.

|  | 2010 | |  |  |  | 2011 |  |  |
| --- | --- | --- | --- | --- | --- | --- | --- | --- |
| Transplant Height† | Plains | | Mississippi Valley | Northeast |  | Plains | Mississippi Valley | Northeast |
| Mean (SD) | 2.114 (0.291) | | 2.055 (0.306) | 1.916 (0.289) |  | 9.853 (2.905) | 9.528 (2.663) | 8.103 (2.720) |
| N (N female) | 59 | | 54 | 36 |  | 62 | 57 | 39 |
| Post-hoc test results | A/B | | B | B/C |  | A | A | B |
|  | 2010 | |  |  |  | 2011 |  |  |
| Flowering Height | Plains | | Mississippi Valley | Northeast |  | Plains | Mississippi Valley | Northeast |
| Mean (SD) | 68.434 (24.678) | | 79.078 (28.409) | 48.181 (34.570) |  | 111.679 (31.436) | 120.244 (28.536) | 90.046 (23.311) |
| N | 59 | | 54 | 36 |  | 62 | 57 | 39 |
| Post-hoc test results | A | | A | B |  | A | A | B |
|  |  | |  |  |  |  |  |  |
| Mature Height | Plains | | Mississippi Valley | Northeast |  | Plains | Mississippi Valley | Northeast |
| Mean (SD) | 114.525 (33.685) | | 134.250 (33.511) | 83.900 (44.614) |  | 156.644 (31.819) | 166.641 (30.958) | 122.805 (32.083) |
| N | 59 | | 52 | 35 |  | 62 | 56 | 39 |
| Post-hoc test results | A | | B | C |  | A | A | B |
|  |  | |  |  |  |  |  |  |
| Mature Branch Number* | Plains | | Mississippi Valley | Northeast |  | Plains | Mississippi Valley | Northeast |
| Mean (SD) | 5.732 (0.959) | | 6.469 (1.420) | 5.473 (1.828) |  | 6.683 (1.032) | 7.536 (0.960) | 7.400 (1.312) |
| N | 59 | | 52 | 35 |  | 62 | 56 | 39 |
| Post-hoc test results | A | | B | A |  | A/B | A/C | A |
|  |  | |  |  |  |  |  |  |
| Length of Longest Mature Branch* | Plains | | Mississippi Valley | Northeast |  | Plains | Mississippi Valley | Northeast |
| Mean (SD) | 6.267 (2.249) | | 6.720 (2.529) | 6.295 (2.460) |  | 9.652 (1.522) | 9.711 (1.797) | 8.949 (1.811) |
| N | 59 | | 52 | 35 |  | 62 | 56 | 39 |
| Post-hoc test results | A | | A | A |  | A | A | A |
| Dry Above-ground Biomass^¶^ | Plains | | Mississippi Valley | Northeast |  | Plains | Mississippi Valley | Northeast |
| Mean (SD) | 0.950 (0.408) | | 1.174 (0.388) | 0.874 (0.427) |  | 1.901 (0.303) | 1.945 (0.311) | 1.717 (0.386) |
| N | 59 | | 52 | 35 |  | 62 | 56 | 39 |
| Post-hoc test results | A | | B | A |  | A | A | B |
|  |  | |  |  |  |  |  |  |
| Days to Flowering | Plains | | Mississippi Valley | Northeast |  | Plains | Mississippi Valley | Northeast |
| Mean (SD) | 72.320 (9.002) | | 73.390 (8.552) | 67.050 (13.544) |  | 58.980 (7.199) | 62.190 (5.518) | 60.050 (9.182) |
| N | 69 | | 66 | 60 |  | 62 | 57 | 39 |
| Post-hoc test results | A | | A/B | A/C |  | A/B | A/C | A |
| †square-root transformed data in 2010  *square-root transformed data  ^¶^log transformed data | |  |  |  |  |  |  |  |
