# Supplementary material for: Agricultural adaptation in the native North American weed waterhemp, Amaranthus tuberculatus (Amaranthaceae)
Source: PLoS One. 2020 Sep 24;15(9):e0238861. doi: 10.1371/journal.pone.0238861 (PMC7514059; doi:10.1371/journal.pone.0238861)
Supplement: S4 Table — The Northeast region has had data from populations 14 and 18 omitted. Significant values at alpha = 0.05 are bold. (DOCX) [file pone.0238861.s009.docx]

**S4 Table.** **Results from general linear models (GLM) or nonparametric Kruskal-Wallis tests of the effect of fixed and random factors on transplant height, flowering height, mature height, mature branch number, length of longest mature branch, dry above-ground biomass, and days to flowering (populations 14 and 18 omitted).** The Northeast region has had data from populations 14 and 18 omitted. Significant values at alpha = 0.05 are bold.

| GLM | 2010 Transplant Height^b^ | |  | | |  | 2011 Transplant Height |  |  |
| --- | --- | --- | --- | --- | --- | --- | --- | --- | --- |
| Factor | df (Hypothesis, Error) | | F ratio | | | P-value | df (Hypothesis, Error) | F ratio | P-value |
| Intercept | 1, 16.099 | | 5101.295 | | | **<0.001** | 1, 13.311 | 627.422 | **<0.001** |
| Region | 2, 14.855 | | 4.988 | | | **0.022** | 2, 13.167 | 2.944 | 0.088 |
| Population (Region) | 13, 241 | | 2.199 | | | **0.010** | 13, 354 | 6.741 | **<0.001** |
| Sex | 1, 241 | | 0.093 | | | 0.761 | 1, 354 | 0.080 | 0.777 |
| GLM | 2010 Flowering Height^a^ | |  | | |  | 2011 Flowering Height^a^ |  |  |
| Factor | df (Hypothesis, Error) | | F ratio | | | P-value | df (Hypothesis, Error) | F ratio | P-value |
| Intercept | 1, 47.925 | | 30.554 | | | **<0.001** | 1, 17.735 | 184.108 | **<0.001** |
| Region | 2, 13.772 | | 2.334 | | | 0.134 | 2, 13.261 | 2.972 | 0.086 |
| Population (Region) | 13, 238 | | 6.065 | | | **<0.001** | 13, 351 | 7.746 | **<0.001** |
| Block | 2, 238 | | 13.689 | | | **<0.001** | 2, 351 | 9.132 | **<0.001** |
| Transplant Height | 1, 238 | | 2.722 | | | 0.100 | 1, 351 | 0.899 | 0.344 |
| Sex | 1, 238 | | 51.503 | | | **<0.001** | 1, 351 | 2.499 | 0.115 |
| GLM | 2010 Mature Height^a^ | |  | | |  | 2011 Mature Height^a^ |  |  |
| Factor | df (Hypothesis, Error) | | F ratio | | | P-value | df (Hypothesis, Error) | F ratio | P-value |
| Corrected Model | 19 | | 6.510 | | | **<0.001** | 19 | 25.917 | **<0.001** |
| Intercept | 1 | | 49.286 | | | **<0.001** | 1 | 529.026 | **<0.001** |
| Region | 2, 232 | | 22.366 | | | **<0.001** | 2, 342 | 24.946 | **<0.001** |
| Population (Region) | 13, 232 | | 3.224 | | | **<0.001** | 13, 342 | 6.504 | **<0.001** |
| Block | 2, 232 | | 10.700 | | | **<0.001** | 2, 342 | 14.226 | **<0.001** |
| Transplant Height | 1, 232 | | 0.061 | | | 0.805 | 1, 342 | 0.679 | 0.410 |
| Sex | 1, 232 | | 12.471 | | | **<0.001** | 1, 342 | 263.333 | **<0.001** |
| GLM | 2010 Mature Branch Number^a,b^ | | |  | |  | 2011 Mature Branch Number^a,b^ |  |  |
| Factor | df (Hypothesis, Error) | | F ratio | | | P-value | df (Hypothesis, Error) | F ratio | P-value |
| Corrected Model | 19 | | 8.770 | | | **<0.001** | 19 | 16.573 | **<0.001** |
| Intercept | 1 | | 86.664 | | | **<0.001** | 1 | 1010.235 | **<0.001** |
| Region | 2, 232 | | 13.500 | | | **<0.001** | 2, 342 | 16.566 | **<0.001** |
| Population (Region) | 13, 232 | | 3.612 | | | **<0.001** | 13, 342 | 4.670 | **<0.001** |
| Block | 2, 232 | | 7.463 | | | **0.001** | 2, 342 | 5.509 | **0.004** |
| Transplant Height | 1, 232 | | 0.975 | | | 0.325 | 1, 342 | 3.649 | 0.057 |
| Sex | 1, 232 | | 84.114 | | | **<0.001** | 1, 342 | 176.205 | **<0.001** |
| GLM | 2010 Length of Longest Mature Branch^a,b^ | |  | | |  | 2011 Length of Longest Mature Branch^a,b^ |  |  |
| Factor | df (Hypothesis, Error) | | F ratio | | | P-value | df (Hypothesis, Error) | F ratio | P-value |
| Corrected Model | 19 | | 1.870 | | | **0.017** | 19 | 16.602 | **<0.001** |
| Intercept | 1 | | 25.047 | | | **<0.001** | 1 | 693.046 | **<0.001** |
| Region | 2, 232 | | 1.260 | | | 0.286 | 2, 342 | 2.166 | 0.116 |
| Population (Region) | 13, 232 | | 1.157 | | | 0.313 | 13, 342 | 3.272 | **<0.001** |
| Block | 2, 232 | | 8.498 | | | **<0.001** | 2, 342 | 24.398 | **<0.001** |
| Transplant Height | 1, 232 | | 0.870 | | | 0.352 | 1, 342 | 0.523 | 0.470 |
| Sex | 1, 232 | | 0.321 | | | 0.571 | 1, 342 | 185.316 | **<0.001** |
| GLM | 2010 Dry Above-ground Biomass^a,c^ | | | |  |  | 2011 Dry Above-ground Biomass^a,c^ |  |  |
| Factor | df (Hypothesis, Error) | | F ratio | | | P-value | df (Hypothesis, Error) | F ratio | P-value |
| Corrected Model | 19 | | 6.615 | | | **<0.001** | 19 | 27.268 | **<0.001** |
| Intercept | 1 | | 23.692 | | | **<0.001** | 1 | 568.713 | **<0.001** |
| Region | 2, 232 | | 8.516 | | | **<0.001** | 2, 342 | 2.094 | 0.125 |
| Population (Region) | 13, 232 | | 2.167 | | | **0.012** | 13, 342 | 4.405 | **<0.001** |
| Block | 2, 232 | | 12.202 | | | **<0.001** | 2, 342 | 28.749 | **<0.001** |
| Transplant Height | 1, 232 | | 0.016 | | | 0.899 | 1, 342 | 0.239 | 0.625 |
| Sex | 1, 232 | | 60.241 | | | **<0.001** | 1, 342 | 353.448 | **<0.001** |
| Kruskal-Wallis Test | 2010 Days to Flowering^d^ | |  | | |  | 2011 Days to Flowering^d^ |  |  |
| Factor | df | | Chi-squared statistic | | | P-value | df | Chi-squared statistic | P-value |
| Region | 2 | | 0.317 | | | 0.853 | 2 | 3.754 | 0.153 |
| Significant values at alpha = 0.05 are bold. | |  |  | | |  |  |  |  |
| ^a^With square-root transformed transplant height as a covariate (2010), or transplant height as a covariate (2011) | | | | | | | | | |
| ^b^Square-root transformed data | | | | | | | | | |
| ^c^Log transformed data  ^d^Categorical data |  | |  | | |  |  |  |  |
