# Supplementary material for: Agricultural adaptation in the native North American weed waterhemp, Amaranthus tuberculatus (Amaranthaceae)
Source: PLoS One. 2020 Sep 24;15(9):e0238861. doi: 10.1371/journal.pone.0238861 (PMC7514059; doi:10.1371/journal.pone.0238861)
Supplement: S5 Table — SD = standard deviation, N = sample size. The Northeast region has had populations 14 and 18 omitted. Letters in the “post-hoc test results” row represent groups that are significantly different (different letters) or are not significantly different (same letters) with alpha = 0.05, as determined by post-hoc tests. (DOCX) [file pone.0238861.s010.docx]

**S5 Table.** **Mean values, standard deviations, and samples sizes for transplant height, flowering height, mature height, mature branch number, length of longest mature branch, dry above-ground biomass, and days to flowering by region (populations 14 and 18 omitted).** SD = standard deviation, N = sample size. The Northeast region has had populations 14 and 18 omitted. Letters in the “post-hoc test results” row represent groups that are significantly different (different letters) or are not significantly different (same letters) with alpha = 0.05, as determined by post-hoc tests.

|  | | 2010 | |  | |  | |  | | 2011 | |  | |  | |
| --- | --- | --- | --- | --- | --- | --- | --- | --- | --- | --- | --- | --- | --- | --- | --- |
| Transplant Height† | | Plains | | Mississippi Valley | | Northeast | |  | | Plains | | Mississippi Valley | | Northeast | |
| Mean (SD) | | 2.080 (0.313) | | 2.084 (0.288) | | 1.892 (0.237) | |  | | 9.824 (3.449) | | 9.980 (2.684) | | 7.852 (2.369) | |
| N (N female) | | 110 | | 103 | | 45 | |  | | 137 | | 153 | | 81 | |
| Post-hoc test results | | A | | A | | B | |  | | A | | A | | B | |
|  | 2010 | |  | |  | |  | | 2011 | |  | |  | |  |
| Flowering Height | Plains | | Mississippi Valley | | Northeast | |  | | Plains | | Mississippi Valley | | Northeast | |  |
| Mean (SD) | 61.273 (27.064) | | 64.338 (36.117) | | 46.118 (34.042) | |  | | 109.794 (31.406) | | 115.985 (31.508) | | 89.165 (23.660) | |  |
| N | 110 | | 103 | | 45 | |  | | 137 | | 153 | | 81 | |  |
| Post-hoc test results | A | | A | | B | |  | | A | | A | | B | |  |
|  |  | |  | |  | |  | |  | |  | |  | |  |
| Mature Height | Plains | | Mississippi Valley | | Northeast | |  | | Plains | | Mississippi Valley | | Northeast | |  |
| Mean (SD) | 108.353 (34.468) | | 124.965 (37.954) | | 86.807 (40.874) | |  | | 130.221 (39.737) | | 132.784 (41.187) | | 104.787 (33.308) | |  |
| N | 109 | | 99 | | 44 | |  | | 135 | | 148 | | 79 | |  |
| Post-hoc test results | A | | B | | C | |  | | A | | A | | B | |  |
|  |  | |  | |  | |  | |  | |  | |  | |  |
| Mature Branch Number* | Plains | | Mississippi Valley | | Northeast | |  | | Plains | | Mississippi Valley | | Northeast | |  |
| Mean (SD) | 5.071 (1.423) | | 5.768 (1.658) | | 4.962 (1.957) | |  | | 5.919 (1.281) | | 6.421 (1.426) | | 6.604 (1.619) | |  |
| N | 109 | | 99 | | 44 | |  | | 135 | | 148 | | 79 | |  |
| Post-hoc test results | A | | B | | A | |  | | A | | B | | B | |  |
|  |  | |  | |  | |  | |  | |  | |  | |  |
| Length of Longest Mature Branch* | Plains | | Mississippi Valley | | Northeast | |  | | Plains | | Mississippi Valley | | Northeast | |  |
| Mean (SD) | 6.149 (2.324) | | 6.632 (2.389) | | 6.521 (2.441) | |  | | 8.149 (2.325) | | 8.249 (2.164) | | 7.932 (1.934) | |  |
| N | 109 | | 99 | | 44 | |  | | 135 | | 148 | | 79 | |  |
| Post-hoc test results | A | | A | | A | |  | | A | | A | | A | |  |
| Dry Above-ground Biomass^¶^ | Plains | | Mississippi Valley | | Northeast | |  | | Plains | | Mississippi Valley | | Northeast | |  |
| Mean (SD) | 0.807 (0.423) | | 0.976 (0.438) | | 0.798 (0.464) | |  | | 1.499 (0.528) | | 1.511 (0.504) | | 1.454 (0.483) | |  |
| N | 109 | | 99 | | 44 | |  | | 135 | | 148 | | 79 | |  |
| Post-hoc test results | A | | B | | A | |  | | A | | A | | A | |  |
|  |  | |  | |  | |  | |  | |  | |  | |  |
| Days to Flowering | Plains | | Mississippi Valley | | Northeast | |  | | Plains | | Mississippi Valley | | Northeast | |  |
| Mean (SD) | 69.490 (10.073) | | 68.940 (11.175) | | 67.100(13.351) | |  | | 58.420 (6.967) | | 59.840 (7.008) | | 59.560 (8.926) | |  |
| N | 135 | | 139 | | 73 | |  | | 137 | | 153 | | 81 | |  |
| Post-hoc test results^ | -- | | -- | | -- | |  | | -- | | -- | | -- | |  |
|  | | | | | | | | | | | | | | |  |
| †square-root transformed data in 2010  *square-root transformed data  ^¶^log transformed data  ^Dunn’s multiple pairwise comparison tests not performed, as region was not significant in Kruskal-Wallis test | | | | | | | | | | | | | | |  |
