# Supplementary material for: Agricultural adaptation in the native North American weed waterhemp, Amaranthus tuberculatus (Amaranthaceae)
Source: PLoS One. 2020 Sep 24;15(9):e0238861. doi: 10.1371/journal.pone.0238861 (PMC7514059; doi:10.1371/journal.pone.0238861)
Supplement: S6 Table — (DOCX) [file pone.0238861.s011.docx]

**S6 Table.** **Pearson correlation matrices for mature plant data, 2010 and 2011 common gardens.**

|  | 2010 |  |  |  |  | 2011 |  |  |  |
| --- | --- | --- | --- | --- | --- | --- | --- | --- | --- |
|  | Mature Height | Mature Branch Number | Length of Longest Mature Branch | Dry Above-ground Biomass |  | Mature Height | Mature Branch Number | Length of Longest Mature Branch | Dry Above-ground Biomass |
| Mature Height | 1.000 |  |  |  |  | 1.000 |  |  |  |
| Mature Branch Number | 0.753 | 1.000 |  |  |  | 0.726 | 1.000 |  |  |
| Length of Longest Mature Branch | 0.381 | 0.309 | 1.000 |  |  | 0.832 | 0.690 | 1.000 |  |
| Dry Above-ground Biomass | 0.556 | 0.558 | 0.699 | 1.000 |  | 0.742 | 0.617 | 0.839 | 1.000 |
