# Supplementary material for: Agricultural adaptation in the native North American weed waterhemp, Amaranthus tuberculatus (Amaranthaceae)
Source: PLoS One. 2020 Sep 24;15(9):e0238861. doi: 10.1371/journal.pone.0238861 (PMC7514059; doi:10.1371/journal.pone.0238861)
Supplement: S7 Table — The Mississippi Valley region has had data from populations 7 and 12 omitted. Significant values at alpha = 0.05 are bold. (DOCX) [file pone.0238861.s012.docx]

**S7 Table. Results from general linear models (GLM) or nonparametric Kruskal-Wallis tests of the effect of fixed and random factors on transplant height, flowering height, mature height, mature branch number, length of longest mature branch, dry above-ground biomass, and days to flowering (populations 7 and 12 omitted).** The Mississippi Valley region has had data from populations 7 and 12 omitted. Significant values at alpha = 0.05 are bold.

| GLM | 2010 Transplant Height^b^ | |  | | |  | 2011 Transplant Height |  |  |  |
| --- | --- | --- | --- | --- | --- | --- | --- | --- | --- | --- |
| Factor | df (Hypothesis, Error) | | F ratio | | | P-value | df (Hypothesis, Error) | F ratio | P-value |  |
| Intercept | 1, 14.691 | | 6440.644 | | | **<0.001** | 1, 13.182 | 711.500 | **<0.001** |  |
| Region | 2, 13.924 | | 9.160 | | | **0.003** | 2, 12.988 | 4.435 | **0.034** |  |
| Population (Region) | 13, 225 | | 1.490 | | | 0.122 | 13, 329 | 5.471 | **<0.001** |  |
| Sex | 1, 225 | | 2.170 | | | 0.142 | 1, 329 | 0.149 | 0.700 |  |
| GLM | 2010 Flowering Height^a^ | |  | | |  | 2011 Flowering Height^a^ |  |  | |
| Factor | df (Hypothesis, Error) | | F ratio | | | P-value | df (Hypothesis, Error) | F ratio | P-value | |
| Intercept | 1, 51.635 | | 26.147 | | | **<0.001** | 1, 22.932 | 169.075 | **<0.001** | |
| Region | 2, 13.390 | | 6.205 | | | **0.012** | 2, 13.116 | 7.644 | **0.006** | |
| Population (Region) | 13, 222 | | 6.450 | | | **<0.001** | 13, 326 | 10.951 | **<0.001** | |
| Block | 2, 222 | | 11.456 | | | **<0.001** | 2, 326 | 7.896 | **<0.001** | |
| Transplant Height | 1, 222 | | 1.119 | | | 0.291 | 1, 326 | 0.114 | 0.763 | |
| Sex | 1, 222 | | 37.919 | | | **<0.001** | 1, 326 | 1.598 | 0.207 | |
| GLM | 2010 Mature Height^a^ | |  | | |  | 2011 Mature Height^a^ |  |  | |
| Factor | df (Hypothesis, Error) | | F ratio | | | P-value | df (Hypothesis, Error) | F ratio | P-value | |
| Corrected Model | 19 | | 8.776 | | | **<0.001** | 19 | 35.448 | **<0.001** | |
| Intercept | 1 | | 52.560 | | | **<0.001** | 1 | 492.348 | **<0.001** | |
| Region | 2, 217 | | 43.116 | | | **0.004** | 2, 316 | 80.894 | **<0.001** | |
| Population (Region) | 13, 217 | | 3.547 | | | **<0.001** | 13, 316 | 10.175 | **<0.001** | |
| Block | 2, 217 | | 10.328 | | | **<0.001** | 2, 316 | 11.410 | **<0.001** | |
| Transplant Height | 1, 217 | | 0.084 | | | 0.773 | 1, 316 | 0.323 | 0.570 | |
| Sex | 1, 217 | | 8.756 | | | **0.003** | 1, 316 | 221.042 | **<0.001** | |
| GLM | 2010 Mature Branch Number^a,b^ | | |  | |  | 2011 Mature Branch Number^a,b^ |  |  | |
| Factor | df (Hypothesis, Error) | | F ratio | | | P-value | df (Hypothesis, Error) | F ratio | P-value | |
| Corrected Model | 19 | | 9.972 | | | **<0.001** | 19 | 17.623 | **<0.001** | |
| Intercept | 1 | | 79.581 | | | **<0.001** | 1 | 936.838 | **<0.001** | |
| Region | 2, 217 | | 24.772 | | | **<0.001** | 2, 316 | 15.086 | **<0.001** | |
| Population (Region) | 13, 217 | | 3.953 | | | **<0.001** | 13, 316 | 7.231 | **<0.001** | |
| Block | 2, 217 | | 5.991 | | | **0.003** | 2, 316 | 4.577 | **0.011** | |
| Transplant Height | 1, 217 | | 0.049 | | | 0.825 | 1, 316 | 4.676 | **0.031** | |
| Sex | 1, 217 | | 84.882 | | | **<0.001** | 1, 316 | 145.941 | **<0.001** | |
| GLM | 2010 Length of Longest Mature Branch^a,b^ | |  | | |  | 2011 Length of Longest Mature Branch^a,b^ |  |  | |
| Factor | df (Hypothesis, Error) | | F ratio | | | P-value | df (Hypothesis, Error) | F ratio | P-value | |
| Corrected Model | 19 | | 1.564 | | | 0.067 | 19 | 23.333 | **<0.001** | |
| Intercept | 1 | | 30.111 | | | **<0.001** | 1 | 625.364 | **<0.001** | |
| Region | 2, 217 | | 0.732 | | | 0.482 | 2, 316 | 24.780 | **<0.001** | |
| Population (Region) | 13, 217 | | 1.023 | | | 0.430 | 13, 316 | 8.384 | **<0.001** | |
| Block | 2, 217 | | 7.482 | | | **0.001** | 2, 316 | 20.750 | **<0.001** | |
| Transplant Height | 1, 217 | | 0.281 | | | 0.596 | 1, 316 | 1.860 | 0.174 | |
| Sex | 1, 217 | | 0.049 | | | 0.824 | 1, 316 | 165.840 | **<0.001** | |
| GLM | 2010 Dry Above-ground Biomass^a,c^ | | | |  |  | 2011 Dry Above-ground Biomass^a,c^ |  |  | |
| Factor | df (Hypothesis, Error) | | F ratio | | | P-value | df (Hypothesis, Error) | F ratio | P-value | |
| Corrected Model | 19 | | 6.794 | | | **<0.001** | 19 | 35.880 | **<0.001** | |
| Intercept | 1 | | 24.502 | | | **<0.001** | 1 | 474.247 | **<0.001** | |
| Region | 2, 217 | | 11.716 | | | **<0.001** | 2, 316 | 29.272 | **<0.001** | |
| Population (Region) | 13, 217 | | 2.722 | | | **0.001** | 13, 316 | 11.872 | **<0.001** | |
| Block | 2, 217 | | 10.869 | | | **<0.001** | 2, 316 | 26.321 | **<0.001** | |
| Transplant Height | 1, 217 | | 0.002 | | | 0.965 | 1, 316 | 0.710 | 0.400 | |
| Sex | 1, 217 | | 51.027 | | | **<0.001** | 1, 316 | 299.811 | **<0.001** | |
| Kruskal-Wallis Test | 2010 Days to Flowering^d^ | |  | | |  | 2011 Days to Flowering^d^ |  |  | |
| Factor | df | | Chi-squared statistic | | | P-value | df | Chi-squared statistic | P-value | |
| Region | 2 | | 22.133 | | | **<0.001** | 2 | 24.014 | **<0.001** | |
| Significant values at alpha = 0.05 are bold. | |  |  | | |  |  |  |  | |
| ^a^With square-root transformed transplant height as a covariate (2010), or transplant height as a covariate (2011) | | | | | | | | | | |
| ^b^Square-root transformed data | | | | | | | | | | |
| ^c^Log transformed data  ^d^Categorical data |  | |  | | |  |  |  |  | |
