# Supplementary material for: Agricultural adaptation in the native North American weed waterhemp, Amaranthus tuberculatus (Amaranthaceae)
Source: PLoS One. 2020 Sep 24;15(9):e0238861. doi: 10.1371/journal.pone.0238861 (PMC7514059; doi:10.1371/journal.pone.0238861)
Supplement: S9 Table — Climatic data for the 2010 common garden (in Eureka, MO) is from the St. Louis International Airport; climatic data for the 2011 common garden (in Oxford, OH) is from the Hamilton-Butler County Regional Airport. Climatic data downloaded from the NOAA National Centers for Environmental Information’s Climate Data Online (http://ncdc.noaa.gov/cdo-web/); soil type data downloaded from the USDA Natural Resources Conservation Service’s Web Soil Survey (http://websoilsurvey.nrcs.usda.gov/app/). (DOCX) [file pone.0238861.s014.docx]

**S9 Table.** **Climatic data and soil type data for 2010 and 2011 common garden locations.** Climatic data for the 2010 common garden (in Eureka, MO) is from the St. Louis International Airport; climatic data for the 2011 common garden (in Oxford, OH) is from the Hamilton-Butler County Regional Airport. Climatic data downloaded from the NOAA National Centers for Environmental Information’s Climate Data Online (http://ncdc.noaa.gov/cdo-web/); soil type data downloaded from the USDA Natural Resources Conservation Service’s Web Soil Survey (http://websoilsurvey.nrcs.usda.gov/app/).

| 2010 Common Garden | Month in 2010 | | | | | |
| --- | --- | --- | --- | --- | --- | --- |
| Climatic Data | May | June | July | August | September | October |
| Mean Maximum Temperature (F) | 77.2 | 89.5 | 91.0 | 91.8 | 81.2 | 74.1 |
| Mean Minimum Temperature (F) | 58.9 | 71.6 | 73.8 | 72.6 | 61.4 | 49.6 |
| Monthly Mean Temperature (F) | 68.1 | 80.6 | 82.4 | 82.2 | 71.3 | 61.9 |
| Days of rain (out of total days) | 15/31 | 13/30 | 17/31 | 6/31 | 13/30 | 5/31 |
| Total Precipitation (in) | 4.64 | 4.04 | 6.69 | 3.62 | 3.73 | 1.06 |
| Soil Type | Fishpot-Urban land-Freeburg complex, 0 to 2 percent slopes, frequently flooded | | | | | |
| 2011 Common Garden | Month in 2011 | | | | | |
| Climatic Data | May | June | July | August | September | October |
| Mean Maximum Temperature (F) | 73.0 | 80.0 | 92.7 | 87.6 | 74.3 | 67.0 |
| Mean Minimum Temperature (F) | 55.0 | 60.0 | 71.1 | 61.7 | 54.9 | 40.9 |
| Monthly Mean Temperature (F) | 64.0 | 71.0 | 81.9 | 74.7 | 64.6 | 54.0 |
| Days of rain (out of total days) | 17/31 | 12/30 | 4/31 | 11/31 | 23/30 | 12/31 |
| Total Precipitation (in) | 6.68 | 3.96 | 0.86 | 1.92 | 7.85 | 3.76 |
| Soil Type | Russell-Miamian silt loams, 2 to 6 percent slope, moderately eroded | | | | | |
