# Supplementary material for: Agricultural adaptation in the native North American weed waterhemp, Amaranthus tuberculatus (Amaranthaceae)
Source: PLoS One. 2020 Sep 24;15(9):e0238861. doi: 10.1371/journal.pone.0238861 (PMC7514059; doi:10.1371/journal.pone.0238861)
Supplement: S1 Methods and Results — (DOCX) [file pone.0238861.s015.docx]

**Materials and methods**

**Study system**

*Amaranthus tuberculatus* sensu lato (including *A. tuberculatus* sensu Sauer, 1955 and *A. rudis* sensu Sauer, 1972), is a native North American herbaceous annual. The species range extends from the Great Plains (roughly as far west as the 100^th^ meridian) eastward to Ohio, and from Louisiana northward to Minnesota, with a northern range boundary in southern Ontario (Fig 1). It is dioecious (and thus obligately outcrossing) and wind-pollinated, with small one-seeded utricle fruits that may be dehiscent or indehiscent. Natural populations of waterhemp are almost always found in wet habitats, especially seasonally flooded riverbanks in the Midwest, but also including creek banks, drainage ditches, lakeshores, and marshy floodplains [1, 2]. Prior to a recent taxonomic study, waterhemp was divided into two species based mainly on utricle dehiscence and geographic range: *A. tuberculatus*, the indehiscent-fruited taxon found to the east of the Mississippi River; and *A. rudis* (earlier misapplied name = *A. tamariscinus*; see [3]), the dehiscent-fruited taxon found most frequently west of the Mississippi River, and commonly considered the "weedy" form of waterhemp [3, 4] (Fig 1). Sauer hypothesized that the two species were brought into contact by the spread of large-scale Midwestern agriculture and the subsequent waterhemp invasion into crop fields in the 20^th^ century [4]. Pratt and Clark [5] found a continuum of morphological and isozyme characters across the range of the more broadly defined *A. tuberculatus*, but some authors still distinguish the two former species as varieties: *A. tuberculatus* var. *tuberculatus* and var. *rudis* [6]. The latter taxonomy is used in this paper, with Pratt and Clark’s species referred to as *A. tuberculatus* sensu lato (s.l.). While the relationship between these morphological varieties and the two genetic subgroups within *A. tuberculatus* s.l. has not been precisely characterized, they do appear to be broadly congruent based on geography.

For the purposes of the common garden experiments, the geographical range was divided into three regions, where populations were hypothesized to have three different levels of adaptation to agricultural environments: the Plains region (including KS, NE, and OK populations in the experiment), the Mississippi Valley region (including MO, IA, and IL), and the Northeast region (including OH, MI, and ON) (Fig 1). Agricultural waterhemp is a serious, economically important pest in northern Missouri, Iowa, and Illinois, whereas as of 2010, it was an opportunistic weed in the Plains region, and was not known to occur agriculturally in most of Ohio (outside of a handful of western counties; J. Stachler, pers. comm.) and had only just begun to invade Ontario [7].

Waterhemp has several growth traits that allow it to compete with crop plants, including discontinuous germination throughout the crop growing season, fast growth rate, and large potential maximum size. Its fitness in crop fields is often measured via some combination of leaf area, branch number, height, and biomass [8]. Several previous experiments testing waterhemp fitness in agricultural environments focused on the impact of waterhemp on soybean yield [9, 10]. Other waterhemp common garden experiments were explicitly designed to test the best methods for controlling waterhemp with herbicides, or to support hypotheses about resistance to various herbicides (e.g., [11-13]). Still others specifically measured waterhemp fitness as a function of emergence date in agricultural plots, with the goal of determining when best to control waterhemp [14, 15].

**Common garden design**

**Seed collection**

In the fall of 2009 and 2010, seeds were collected from populations across the range of *A. tuberculatus* s.l. for use in common garden experiments in two locations: Missouri (summer 2010) and Ohio (summer 2011). Waterhemp is a common species in naturally disturbed habitats throughout the central U.S. and Ontario, Canada, and no permits are required for its collection in public spaces, where these seeds were obtained. For both common garden experiments, six populations from each of the three different geographic regions (see above) were selected for the experiment (Fig 1). Almost all populations were sampled from natural habitats rather than agricultural fields, with the exception of one Iowa population (Population 7), and one Illinois population (Population 12), which were included to maximize geographical representation. Population structure analyses indicate there is no genetic differentiation between agricultural and natural populations on the geographical scale of central U.S. states [16]; however, to test for a possible effect of including these agricultural populations, data were analyzed with and without Populations 7 and 12 (see Data analysis, below). Seeds from the two Iowa populations were obtained from the USDA GRIN database: they were originally collected in 1989 and 1996 by D. Pratt, and seed stocks have been maintained by the USDA without being grown out since collection (D. Brenner, pers. comm.).

For the two years of the common garden experiments (2010, 2011), the same source populations were used to represent the Plains region and the Mississippi Valley region. For the Northeast region, two newly-collected Ohio populations were included in 2011 (and two previous Northeastern populations from 2010 were dropped) to represent the portion of the state where agricultural fields have been invaded by waterhemp, and to attempt to correct for possible confounding of latitude of origin with agricultural adaptation (see Table 1 and Fig 1). Voucher specimens for each population were deposited at the Missouri Botanical Garden (MO) herbarium. By morphological criteria, the Plains and Mississippi Valley populations were mostly *A. tuberculatus* var. *rudis*, whereas the Northeast populations were mostly *A. tuberculatus* var. *tuberculatus* (Table 1).

**Common garden setup**

Seeds from 10 female plants per population were stored at room temperature with silica gel or frozen at -20ºC until three to four months before the common garden experiments. At this point, 16 seeds were randomly selected from each individual (parent) and placed on a damp paper towel inside a ziploc bag, stored at 4ºC for three to four months. This stratification procedure mimics the natural winter stratification of shallowly-buried seeds in *A. tuberculatus* habitats. If 16 seeds were not available for a particular parent, as many as were available were used, and supplementary seeds were stratified from another individual of the same population. Obtaining adequate sample sizes was also the rationale for combining seeds from two proximate Northeast geographical locations into a single experimental population in two instances (see Table 1; population 17 in 2010, population 18 in both years). Seeds for the 2010 common garden were stratified from Feb. 1 to May 18, and seeds from the 2011 garden were stratified from Feb. 14 to June 9. Planting was timed to coincide with soybean planting in both years.

Eight randomly-selected seeds per parent were planted in 98-well flats, with two seeds/labeled well, in the Washington University greenhouse. The newly-planted seeds were placed on a mist bench for 1-2 days to facilitate germination, and then moved to a warm sunny bench in the greenhouse. The plants were thinned to one per well soon after germination, and poor germination for seeds from a particular parent was compensated with seedlings from another parent from the same population, for an average of ~3 seedlings per maternal plant. There is some evidence in the genus *Amaranthus* that there are strong maternal effects on seed dormancy/germination [17, 18]; therefore, the height of each seedling was recorded just prior to transplanting, to use as a control for maternal effects on early growth. Just prior to transplanting, seedlings from the same parent were sorted into three groups, so that each group had one seedling/parent and 10 seedlings/population, for a total of 180 seedlings per group. Then, seedlings were randomly assigned a number from 1-540, and the seedlings were arranged in numerical order in the sets of 180, one for each block in the common garden.

Between seed stratification and transplanting, the common garden plots were prepared; see S9 Table for climate and soil type data for the two common garden locations. In 2010 (the Missouri common garden), three “old field” sites at Washington University’s Tyson Research Center (Eureka, MO: 38.526071ºN, 90.561748ºW) were chosen on the basis of their similarity and suitability for soybeans. These three blocks measured 7 x 10 m, and were tilled with a rotary cultivator on 5 May. RoundUp Ready soybeans (Asgrow RR3830, Monsanto, St. Louis, Missouri, USA) were planted by hand 1 inch (2.54 cm) deep in rows between 19-26 May, with 19 rows/block spaced 0.5 m apart, and ~150 soybeans/row (4-5 cm apart; 160,000 seeds/acre), according to standard recommendations in UM-Extension publications [19]. Polypropylene deer fencing was placed around each block on 15-16 June. The ~3-to-4-week-old waterhemp plants were transplanted into the soybean blocks when the soybeans were at the first node (V1) stage from 16-19 June, and immediately watered for establishment by hand. The waterhemp rows were 5 meters long (1 m from the fence on either side horizontally and 1.5 m from the fence vertically), and placed between soybean rows (20 cm from each soybean row and 50 cm from the next waterhemp row), with individuals spaced 40 cm apart in the row to avoid intraspecific competition. Each block had 13 rows of waterhemp with 13 plants per row, and a 14th row with 11 plants. The blocks were hand weeded throughout the growing season to remove all plants other than soybeans and waterhemp.

In 2011 (the Ohio common garden), field plots were established at Miami University’s Ecology Research Center (ERC), in Oxford, OH (39.531484ºN, 84.722837ºW). The experiment was designed to replicate as nearly as possible the 2010 Missouri common garden, with some notable exceptions. The three Oxford blocks were randomly placed in a single 27 x 92 m soybean field, for most convenient mechanical soybean planting. Again, blocks measured 7 x 10 m, and were tilled and drill-planted with RoundUp Ready soybeans (Genuity Star RR3404, Monsanto, St. Louis, Missouri, USA) on 8 June, in rows 0.5 m apart (160,000 seeds/acre). Because of an unusually wet spring in Ohio, soybean planting was delayed compared to the previous year. Deer herbivory on the emerging soybeans was severe, necessitating soybean replanting on 25 June. Because of this, waterhemp transplanting was delayed and thus waterhemp seedlings were kept in the Washington University greenhouse for 16 more growing-degree days than in 2010, making it necessary to move the seedlings into a larger pot size (24-well flats) on 27 June to prevent them from becoming pot-bound and stunted. The ~3-to-4-week old waterhemp seedlings were transplanted into the common garden blocks from 6-8 July, and immediately watered for establishment by hand. A four-wire electric fence was installed around the entire field on 27 June, and polypropylene deer fencing was installed around individual blocks on 8 July. The spatial positioning of waterhemp rows and individuals was the same as for the Missouri blocks, except that the double soybean planting led to essentially random spacing of soybeans with respect to waterhemp rows. The blocks were hand weeded throughout the growing season.

**Plant measurements, 2010**

Starting a few days after transplanting and every week thereafter, plant survivorship in the Missouri common garden blocks was recorded. Flowering started on 29 June, and flowering start date, flowering plant height, and sex of the plant was recorded from 29 June to 19 August, every 5-9 days. Sex determination in waterhemp is clear-cut, with only male or only female flowers on each plant, and with occasional neuter (sterile) plants occurring in natural populations (K. Waselkov, pers. obs.). We observed six neuter plants in our experiments and excluded these from analyses. An open flower on a male or female plant was taken as the start of flowering, and days to flowering was measured as the number of days between planting and the start of flowering. Mature plant measurements were taken when ~75% of flowers were open (for male plants, which grow very little once flowering begins) or ~75% of flowers had set seed (for female plants, which continue to grow after flowering begins). Mature height, number of branches off the main stem, and length of the longest primary branch were recorded for each waterhemp plant. These measurements were taken between 13 August and 5 October as plants matured asynchronously, approximately every 2-3 weeks (except Block 1, for which measurements were recorded on 13 August, 19 August, 29 August, and 2 October).

Immediately after mature measurements were taken, the plant’s above-ground biomass was removed at ground level, placed in a brown paper bag, and stored at Washington University. A Conviron plant growth chamber (PGW36 model, Conviron, Winnipeg, Manitoba, Canada) in the Washington University greenhouse was used to dry the plants. The growth chamber was set at 37-39ºC, 20-26% relative humidity, and lamps at 44 watts/m^2^. Batches of bags were left in the chamber for 9-15 days, at which time each bagged plant was weighed on an electronic scale (preliminary experiments established that dry weight stabilized at 9 days). Dried above-ground biomass measurements were recorded to the nearest 0.01 gram, and the average weight of five empty bags was subtracted from the raw biomass measurement. The number or weight of seeds per plant was not quantified, because waterhemp plants in crop fields can produce up to a million seeds each [20], and seed production is not confined to discrete seed heads but spread over the entire plant. Thus, seed production was judged impractical to measure, and dry above-ground biomass was taken to be the most feasible proxy measure of fecundity. Waterhemp biomass and female fecundity (seed production) have been shown to be closely correlated [14, 21].

**Plant measurements, 2011**

The same procedures were followed for the Ohio common garden, except for modifications described below. Because the geographical area around Oxford, OH did not yet have a problem with agricultural waterhemp in 2011, procedures were implemented to contain gene flow from the experimental waterhemp into surrounding agricultural fields and/or nearby riverbank populations. Therefore, plants in the Ohio common garden were monitored much more frequently than in the Missouri common garden: survival, flowering start date, flowering plant height, and sex of the plant were recorded every 2-3 days, from 11 July-19 August. Furthermore, to prevent the pollen from being dispersed by wind, male plants were measured for mature data and harvested as soon as their first flower opened. As males grow very little after flowering begins, this difference is unlikely to have influenced inferences (see Results). Female plants were measured for mature height, number of branches, and length of the longest branch and harvested at approximately the same point as in the Missouri common garden, before many seeds/fruits could drop from the plant. Male plants were harvested every two-three days from 15 June to 2 September. Female plants were harvested every 2-3 weeks from 2 September to 12 October. Dried biomass for these plants was measured in exactly the same way as for the Missouri garden.

**Data analysis**

All plant data were analyzed using IBM SPSS Statistics 1.0.0.1213 (IBM Corp., 2018). Each year’s data were analyzed separately, as was each measured response variable. First, all continuous data were tested for normality using the Shapiro-Wilk test. If the data were not normal, they were either log_10_ transformed or square-root transformed. Analyses were run with and without outliers (detected using SPSS box plots). Nonparametric tests were used for ordinal data (days to flowering).

A univariate general linear model (GLM) or the equivalent nonparametric test was used to analyze each dataset, including days to flowering, height at transplantation, flowering plant height, mature plant height, branch number, length of the longest branch, and dry above-ground biomass. Levene’s test of equality of error variances was conducted for all analyses of continuous dependent variables. Additionally, a repeated-measures general linear model was used to analyze (untransformed) height over time, and Greenhouse-Geisser test values are reported to correct for significant deviations from sphericity. Multivariate GLM was also used to analyze mature plant height, branch number, longest branch length, and dry above-ground biomass together, because of the correlation of these mature plant measurements (moderate positive correlation (0.3-0.85) verified with a Pearson’s correlation matrix). Box’s *M* test was used to check for homogeneity of covariance matrices between regions in the multivariate GLMs; if this test was significant, results of Pillai’s Trace tests are reported, as this statistic is robust to violations of multivariate normality and the homogeneity of variance-covariance matrices assumption. Height at transplantation was included as a covariate in all general linear models (with the exception of the repeated measures GLM for height, where it was the first time point in the dataset), to control for maternal effects. The fixed factors in each GLM were geographical region of origin (Plains, Mississippi Valley, and Northeast) and sex of the plant. The random factors were block and population nested within region, and the intercept was included in the model. Interactions between sex, region, and block were included in the models initially, but were omitted for the final analyses as these interaction terms never had a significant effect on the dependent variables (results not shown). For significant results for continuous dependent variables, post-hoc Tukey HSD tests were used to determine whether means were significantly different between each pair of regions and populations. When the results of Levene’s test were significant for continuous data, or when a covariate was included, pairwise comparison of estimated marginal means (with Bonferroni correction of significance values) was performed in lieu of Tukey HSD tests. Dunn’s multiple comparison tests with Bonferroni correction of significance values were performed post hoc for ordinal data.

Because the growing conditions differed substantially between years, the datasets from Missouri and Ohio common gardens were analyzed separately (with no attempt made to combine data across years). For the Missouri common garden, data were analyzed with and without the inclusion of plants that started to show inflorescence development before transplantation, or that were dead when mature measurements were taken (due to the spacing between mature data collection points). For the Ohio common garden, data were analyzed with and without plants infested with ash-gray leaf bugs (*Piesma cinerea* Say) (which appeared to have stunted plant growth; K. Waselkov, pers. obs.). Additionally, to rule out any confounding factors introduced by harvesting the Ohio male plants earlier than the Missouri males, only female data were analyzed for both common gardens and compared to the full data set. To rule out possible bias introduced by including the two agricultural populations (7 and 12), data were analyzed with and without these populations included. Finally, to further assess potential outlier effects of high latitude populations on the response variables, all analyses were run with the omission of data from populations 14 and 18 (the two populations collected at the highest latitudes, both found in the Northeast region) and compared to the full data set.

**Results**

**Agricultural populations**

When the two populations that were originally collected from agricultural habitats (Populations 7 and 12) were omitted from the data analyses, the results were qualitatively almost exactly the same as those based on the full data set (S7 and S8 Tables). A few posthoc relationships change in significance when these populations are omitted, including Mississippi Valley plants becoming significantly taller than Plains plants at flowering in 2010 and 2011, and at maturity in 2011, and Mississippi Valley plants flowering significantly later than Plains and Northeastern plants in both years (vs. only later than Northeastern plants in the full dataset analyses). Thus, it can be inferred that the inclusion of these two populations from agricultural habitats affected the results to a very minor extent, and actually made the results less significantly different between regions, rather than more different.

**References**

1. Mosyakin S, Robertson KR. *Amaranthus*. In: Flora of North America Editorial Committee, editors. Flora of North America North of Mexico, Vol. 4. Magnoliophyta: Caryophyllidae, Pt. 1. New York: Oxford University Press; 2003. pp. 410–435.
2. Sauer JD. Revision of the dioecious amaranths. Madroño. 1955;13: 5–46.
3. Sauer JD. The dioecious amaranths: a new species name and major range extensions. Madroño. 1972;21: 427–434.
4. Sauer JD. Recent migration and evolution of the dioecious amaranths. Evolution. 1957;11: 11–31.
5. Pratt DB, Clark LG. *Amaranthus rudis* and *A. tuberculatus*, one species or two? J Torrey Bot Soc. 2001;128: 282–296.
6. Costea M, Tardif FJ. Conspectus and notes on the genus *Amaranthus* in Canada. Rhodora. 2003;105: 260–281.
7. Schryver MG, Soltani N, Hooker DC, Robinson DE, Tranel PJ, Sikkema PH. Glyphosate-resistant waterhemp (*Amaranthus tuberculatus* var. *rudis*) in Ontario, Canada. Can J Plant Sci. 2017;97: 1057–1067.
8. Horak MJ, Loughin TM. Growth analysis of four *Amaranthus* species. Weed Sci. 2000;48: 347–355.
9. Hager AG, Wax LM, Stoller EW, Bollero GA. Common waterhemp (*Amaranthus rudis*) interference in soybean. Weed Sci. 2002;50: 607–610.
10. Steckel LE, Sprague CL. Late-season common waterhemp (*Amaranthus rudis*) interference in narrow- and wide-row soybean. Weed Technol. 2004;18: 947–952.
11. Steckel LE, Sprague CL, Hager AG. Common waterhemp (*Amaranthus rudis*) control in corn (*Zea mays*) with single preemergence and sequential applications of residual herbicides. Weed Technol. 2002;16: 755–761.
12. Legleiter TR, Bradley KW, Massey RE. Glyphosate-resistant waterhemp (*Amaranthus rudis*) control and economic returns with herbicide programs in soybean. Weed Technol. 2009;23: 54–61.
13. Evans CM, Strom SA, Riechers DE, Davis AS, Tranel PJ, Hager AG. Characterization of a waterhemp (*Amaranthus tuberculatus*) population from Illinois resistant to herbicides from five site-of-action groups. Weed Technol. 2019;33: 400–410.
14. Hartzler RG, Battles BA, Nordby D. Effect of common waterhemp (*Amaranthus rudis*) emergence date on growth and fecundity in soybean. Weed Sci. 2004;52: 242–245.
15. Nordby DE, Hartzler RG. Influence of corn on common waterhemp (*Amaranthus rudis*) growth and fecundity. Weed Sci. 2004;52: 255–259.
16. Waselkov KW, Olsen KM. Population genetics and origin of the native North American agricultural weed waterhemp (*Amaranthus tuberculatus*; Amaranthaceae). Am J Bot. 2014;101: 1726–1736.
17. Costea M, Weaver S, Tardif FJ. The biology of Canadian weeds. 130. *Amaranthus retroflexus* L., *A. powellii* S. Watson and *A. hybridus* L. Can J Plant Sci. 2004;84: 631–668.
18. Jha P. Biology and ecology of Palmer amaranth (*Amaranthus palmeri*). Ph.D. Dissertation, Clemson University. 2008. Available from: <https://tigerprints.clemson.edu/all_dissertations/296>.
19. Helsel ZR, Minor HC. Soybean production in Missouri. Publication G4410. Columbia: University of Missouri Extension; 1993. Available from: <http://extension2.missouri.edu/g4410>.
20. Steckel LE. The dioecious *Amaranthus* spp.: here to stay. Weed Technol. 2007;21: 567–570.
21. Menalled FD, Liebman M, Buhler DD. Impact of composted swine manure and tillage on common waterhemp (*Amaranthus rudis*) competition with soybean. Weed Sci. 2004;52: 605–613.
